# Elucidating Zeolite Channel Geometry–Reaction Intermediate Relationships for the Methanol‐to‐Hydrocarbon Process

**DOI:** 10.1002/anie.202009139

**Published:** 2020-09-11

**Authors:** Donglong Fu, Alessandra Lucini Paioni, Cheng Lian, Onno van der Heijden, Marc Baldus, Bert M. Weckhuysen

**Affiliations:** ^1^ Inorganic Chemistry and Catalysis Debye Institute for Nanomaterials Science Utrecht University 3584 CG Utrecht The Netherlands; ^2^ NMR Spectroscopy Bijvoet Centre for Biomolecular Research Utrecht University Padualaan 8 3584 CH Utrecht The Netherlands; ^3^ Institute for Theoretical Physics, Utrecht University Princetonplein 5 3584 CC Utrecht The Netherlands

**Keywords:** arenes, olefins, NMR spectroscopy, reaction mechanisms, zeolites

## Abstract

The chemical industry has exploited zeolite shape selectivity for more than 50 years, yet our fundamental understanding remains incomplete. Herein, the zeolite channel geometry–reactive intermediate relationships are studied in detail using anisotropic zeolite ZSM‐5 crystals for the methanol‐to‐hydrocarbon (MTH) process, and advanced magic‐angle spinning solid‐state NMR (ssNMR) spectroscopy. The utilization of anisotropic ZSM‐5 crystals enabled the preferential formation of reaction intermediates in single‐orientation zeolite channels, as revealed by molecular dynamics simulations and in situ UV/Vis diffuse‐reflectance spectroscopy. The ssNMR results show that the slightly more constrained sinusoidal zeolite channels favor the olefin cycle by promoting the homologation of alkanes, whereas the more extended straight zeolite channels facilitate the aromatic cycle with a higher degree of alkylation of aromatics. Dynamic nuclear polarization experiments further indicate the preferential formation of heavy aromatics at the zeolite surface dominated by the sinusoidal channels, providing further insight into catalyst deactivation.

## Graphical abstract

The relationships between zeolite channel geometries and reaction intermediates are reported here. Anisotropic zeolite ZSM‐5 crystals were used to enable the preferential formation of reaction intermediates in single‐orientation zeolite channels for the methanol‐to‐hydrocarbon (MTH) process. Advanced solid‐state NMR spectroscopy showed that the more constrained sinusoidal zeolite channels favor the olefin cycle, whereas the more extended straight zeolite channels facilitate the aromatic cycle.

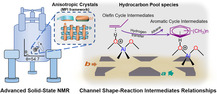

## Introduction

The shape or topology of internal pore structures of zeolites strongly affects the product selectivity by permitting the configuration of different reactants, intermediates or products.[^1^, ^2^, ^3^] This contributes to highly cost‐effective processes with well‐defined product distributions and has been applied by the chemical industry for more than 50 years. One of the prototypical cases is the already commercialized methanol‐to‐hydrocarbon (MTH) process, which converts biomass‐ and municipal waste‐ or coal‐derived methanol into a range of products from olefins to gasolines, depending on the framework topology of the zeolite material chosen.[^4^, ^5^, ^6^, ^7^] As a result, significant research efforts have been focused on elucidating the influence of zeolite topology, that is, channels, cages and cavities, on the ultimate product distributions and coking behaviors.[^8^, ^9^, ^10^, ^11^, ^12^, ^13^, ^14^, ^15^, ^16^, ^17^, ^18^, ^19^, ^20^, ^21^, ^22^, ^23^] Yet, no direct experimental evidence has been presented for the effect of channel geometry on the reaction intermediates.

**Scheme 1:**
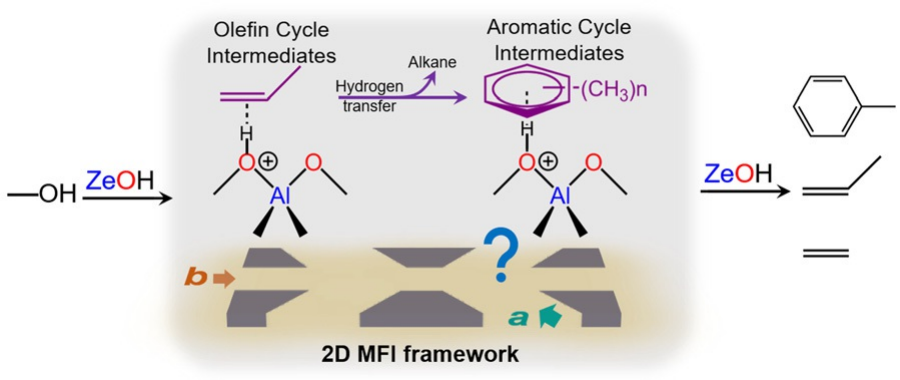
Schematic illustration of the methanol‐to‐hydrocarbon (MTH) process catalyzed by zeolite ZSM‐5 with the MFI framework, consisting of sinusoidal and straight channels. The reaction follows the hydrocarbon pool (HCP) mechanism with an olefinic and an aromatic cycle running simultaneously.

There is a consensus that the MTH reaction proceeds through a dual cycle hydrocarbon pool (HCP) mechanism (Scheme 1), which consists of two interdependent reaction cycles based on aromatic and olefinic species, that is, aromatic and olefin cycles, respectively.[^24^, ^25^, ^26^, ^27^] These aromatic cycle species are repeatedly methylated and subsequently split off light alkenes, contributing to the production of ethylene. The olefin cycle involving methylation and cracking of olefins primarily produces larger alkenes. The confinement effect imposed by zeolite channels often leads to host‐guest interactions between the zeolite framework host and the HCP species guest. Thus, Haw et al. extended the HCP mechanism and proposed that an “hybrid” inorganic‐organic material of “supramolecular nature” consisting of the inorganic zeolite and the organic hydrocarbon pool can be viewed as the active MTH catalyst.[^24^, ^28^, ^29^] The Deng and Xu group recently identified this supramolecular reaction center (SMC)^[30]^ and demonstrated that the pore size and shape of zeolites have prominent impact on the host–guest interactions and the reactivity of the SMC during the MTH process.^[31]^ Further, the research from our group, as well as from the Gascon and van Speybroeck groups, highlighted the complexity and significance of the host‐guest chemistry during the MTH process.[^32^, ^33^, ^34^, ^35^, ^36^, ^37^] Recently, we have shown distinct product distributions and coking behaviors for zeolite channels with different geometries.^[38]^ These findings altogether motivated us to comprehensively scrutinize the distinctive host‐guest chemistry exclusively between zeolite channel geometry and reaction intermediates (Scheme 1). This information is crucial to the design of catalyst materials with maximized product yields and minimized costs.[^39^, ^40^]

Herein, the host‐guest chemistry between single oriented zeolite channels and reaction intermediates for the MTH process has been examined over zeolite ZSM‐5 (possessing the MFI framework topology; see Figures S5a and S5b in the Supporting Information), which is one of the most studied zeolites in both industry and academia. We have utilized anisotropic zeolite ZSM‐5 crystals (Figure S5), with different channel orientations dominating the surface, and demonstrated the preferential formation of reaction intermediates in single oriented, that is, surface dominating, zeolite channels using molecular dynamics (MD) simulations and in situ UV/Vis diffuse‐reflectance spectroscopy (DRS). Furthermore, complementary solid‐state NMR (ssNMR, Figure S4) magnetization transfer schemes were applied to separate reactive MTH intermediates based on their differential mobility^[41]^ over the anisotropic zeolite ZSM‐5 crystals. Finally, we have applied dynamic nuclear polarization (DNP)[^36^, ^42^, ^43^, ^44^, ^45^, ^46^] to improve the sensitivity and examine the spatial distribution of the hydrocarbons within the MTH‐reacted materials.

## Results and Discussion

### Preparation of ^13^C MTH‐Reacted Anisotropic Zeolite Crystals

**Figure 1:**
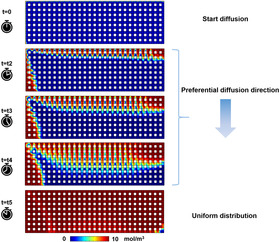
Time‐dependent concentration profiles in the 2D pore network model. Note that diffusion starts at t0. The results from t1 to t4 suggest that methanol molecules preferentially diffuse into the channels with short dimension (SD), with the primary diffusion direction indicated by the arrow. Long time diffusion will result in uniform distribution (t=t5) of methanol molecules in both channel orientations.

Anisotropic zeolite ZSM‐5 crystals with different channels dominating the surface have been synthesized and applied for investigating the impact of zeolite channel geometry on the reaction intermediates for the MTH process. These materials exhibit comparable physicochemical properties, as demonstrated by scanning electron microscope (SEM, Figures S5c–d) as well as MAS ^29^Si and ^27^Al ssNMR (Figures S6 and S7, Table S3).^[47]^ Importantly, as shown in Figure 1, qualitative analysis of the diffusion behaviors in zeolite channels using molecular dynamics (MD) simulations exhibited that the channels with short dimensions (SD) were preferentially filled with methanol molecules at the early stage of diffusion, although both channel dimensions were equivalently filled with the molecules eventually (t=t5 in Figure 1). Furthermore, no apparent diffusion of the molecules from the SD channels into the channels in long dimensions (LD) was observed (Figure 1 and movie S1). These results suggest that the molecules will enrich the SD channels at the beginning of the reactions. Therefore, zeolites with hydrocarbons preferentially formed in single oriented channels can be potentially prepared by performing a short contact time MTH reaction over the anisotropic zeolite crystals with different morphologies.

**Figure 2:**
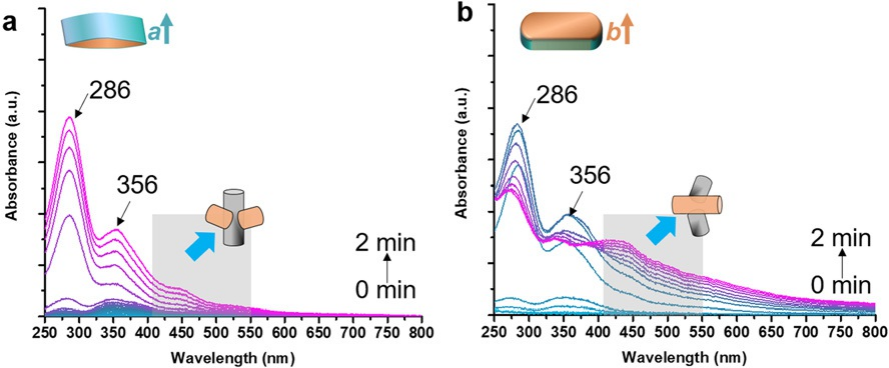
In situ UV/Vis diffuse‐reflectance spectra (DRS) of the a) *a*‐oriented and b) *b*‐oriented zeolite ZSM‐5 crystals during 2 min of the ^13^C labeled MTH process at 623 K.

^13^C‐labeled MTH reactions were monitored using in situ UV/Vis DRS to track the reaction locations over anisotropic zeolite crystals.[^38^, ^48^] The observed bands at ca. 286 and 356 nm are attributed to polyalkyl‐substituted cyclopentadienium ion with four or five alkyl groups and methylbenzenium ions with up to four methyl groups, respectively.^[49]^ Previously, we have demonstrated that hydrocarbon species with absorbance band in the range of ca. 410–560 nm are primarily formed in the straight zeolite channels.^[38]^ Here, these species were used as a marker to locate the reactions within zeolite channels in the anisotropic zeolite ZSM‐5 crystals. As shown in Figure 2, low signal of UV/Vis absorbance in the range of ca. 410–560 nm was observed for the *a*‐oriented zeolite ZSM‐5 crystals during the first 2 min MTH reactions. Conversely, fast evolution and strong absorbance from HCP species were meanwhile observed in the same range for the *b*‐oriented analogue. Taken together with the MD simulations, these results demonstrated that the MTH reactions primarily occurred within the sinusoidal and straight channels in the *a*‐oriented and *b*‐oriented, respectively, ZSM‐5 crystals in the initial stage (that is, 2 min) of the reaction.

### Impact of Channel Geometries on Reaction intermediates

**Figure 3:**
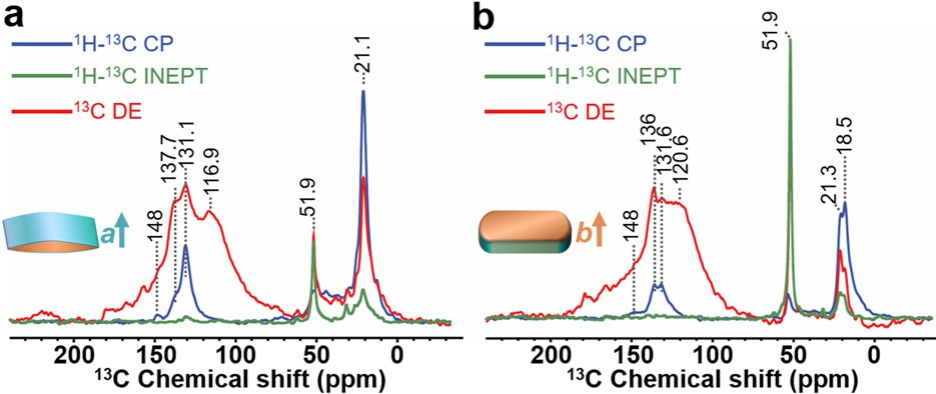
Magic‐angle spinning (MAS) solid‐state nuclear magnetic resonance (ssNMR) spectra of the trapped MTH species in the a) *a*‐oriented and b) *b*‐oriented zeolite crystals. 1D ^1^H‐^13^C cross‐polarization (CP, blue) to probe rigid molecules, ^1^H‐^13^C insensitive nuclei enhanced by polarization transfer (INEPT, green) to probe mobile molecules and ^13^C direct excitation (DE, red) to probe all chemical species, including those that exhibit intermediate dynamics.

Subsequently, ssNMR experiments were conducted over the ^13^C‐MTH‐reacted anisotropic zeolite crystals with hydrocarbons preferentially formed within single oriented zeolite channels. The use of isotope‐enriched methanol not only drastically enhanced the ssNMR signal, but also allowed us to accurately determine the molecular structures of zeolite‐trapped hydrocarbons in different environments using multidimensional ssNMR correlation experiments.[^33^, ^50^] As previously demonstrated, species with high or low mobility can be distinguished by applying either through‐bond (scalar based interactions such as in INEPT, that is, insensitive nuclei enhanced by polarization transfer)^[51]^ or through‐space (dipolar transfer such as in CP, that is, cross polarization,)^[52]^ magnetization transfer schemes, respectively.^[53]^ Thus, both mobile (that is, molecules/groups with fast tumbling or rotation) and rigid (that is, molecules physiosorbed in/on zeolite) versions of zeolite trapped organics after the MTH reaction could be identified. Additionally, direct excitation (DE) experiments were performed to excite all chemical species,^[41]^ including those with intermediate dynamics (for which either an INEPT or CP transfer would be less efficient). In the 1D ^13^C‐spectra (Figure 3) two main features were recognized in both MTH‐reacted *a*‐oriented and *b*‐oriented ZSM‐5 crystals: (i) 9–55 ppm aliphatic moieties and methyl groups, (ii) 110–155 ppm (methylated) aromatic/olefinic groups. It should be noted that the absence of the carbocation form of the MTH intermediates, that is, cyclopentenyl cations and benzenium ions,[^54^, ^55^] in the ssNMR spectra is due to the low quantity of these species after the short MTH reaction time of 2 min. Overall, the 1D ssNMR results in Figure 3 show that the olefinic/aromatic species are either immobilized (detected by cross‐polarization, CP) or exhibit restricted mobility (visible in the direct excitation, DE). In contrast, mobile molecules (visible in scalar‐based INEPT experiments^[51]^) are predominantly saturated/aliphatic in nature. Moreover, the broadening of the ^13^C DE spectra is due to fast relaxing species with intermediate dynamics.

**Figure 4:**
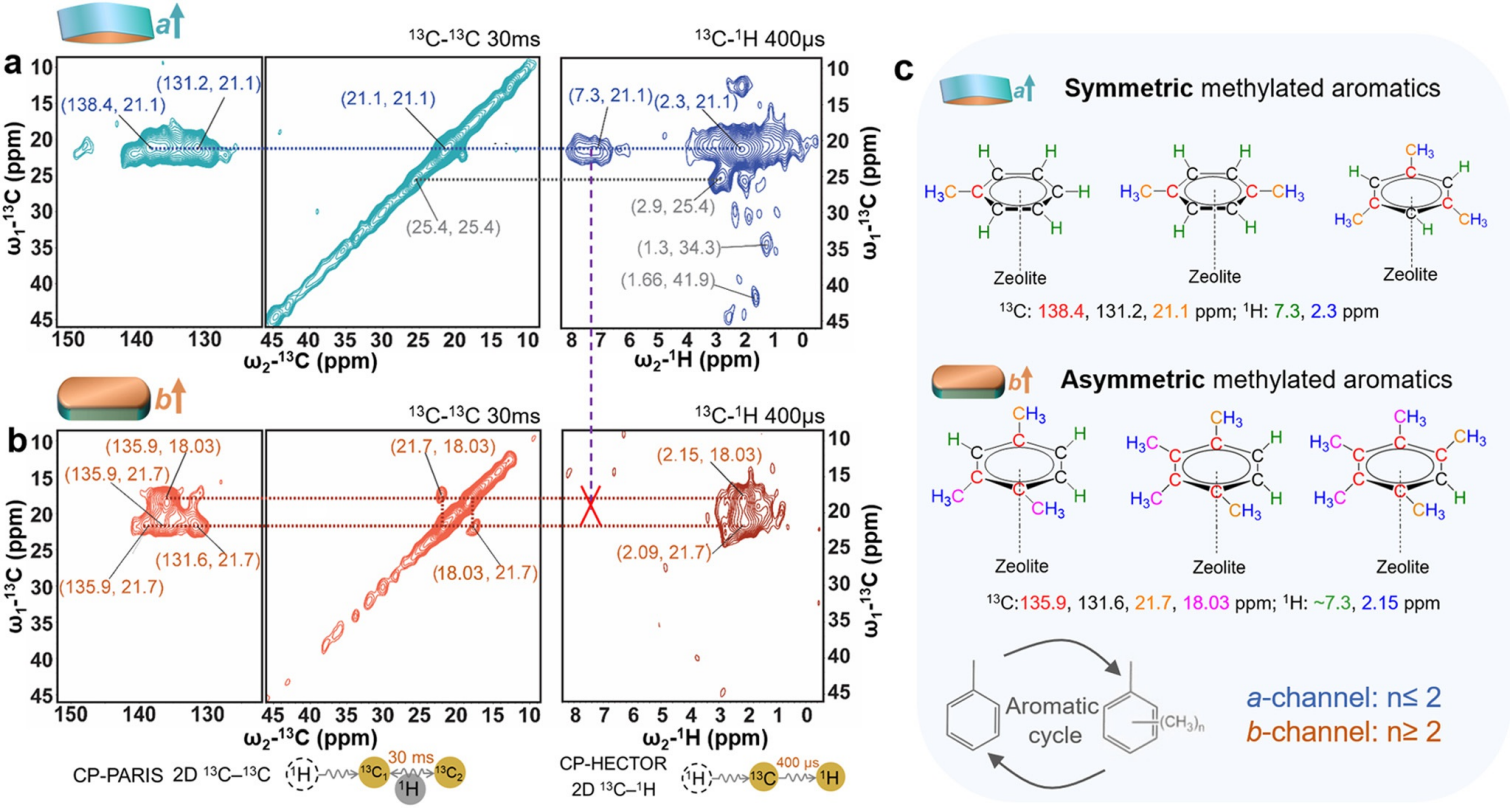
2D ^13^C‐^13^C and ^13^C‐^1^H MAS ssNMR spectra of the rigid methylated aromatics molecules in the a) *a*‐oriented and b) *b*‐oriented zeolite ZSM‐5 crystals after 2 min of the MTH process at 623 K. c) Assignment of the observed hydrocarbons trapped within the anisotropic zeolite crystals. The spectra were recorded at 290 K, using 16 kHz MAS. For the ^13^C‐^13^C correlation experiment, polarization of ^13^C spins was achieved by initial CP and a 30 ms phase‐alternated recoupling irradiation Scheme (PARIS) mixing period.^[30]^ A CP contact time of 400 μs was used for the ^13^C‐^1^H spectra. Resonances colored in gray in the top panel show species that are present only in the *a*‐oriented ZSM‐5 crystals.

**Figure 5:**
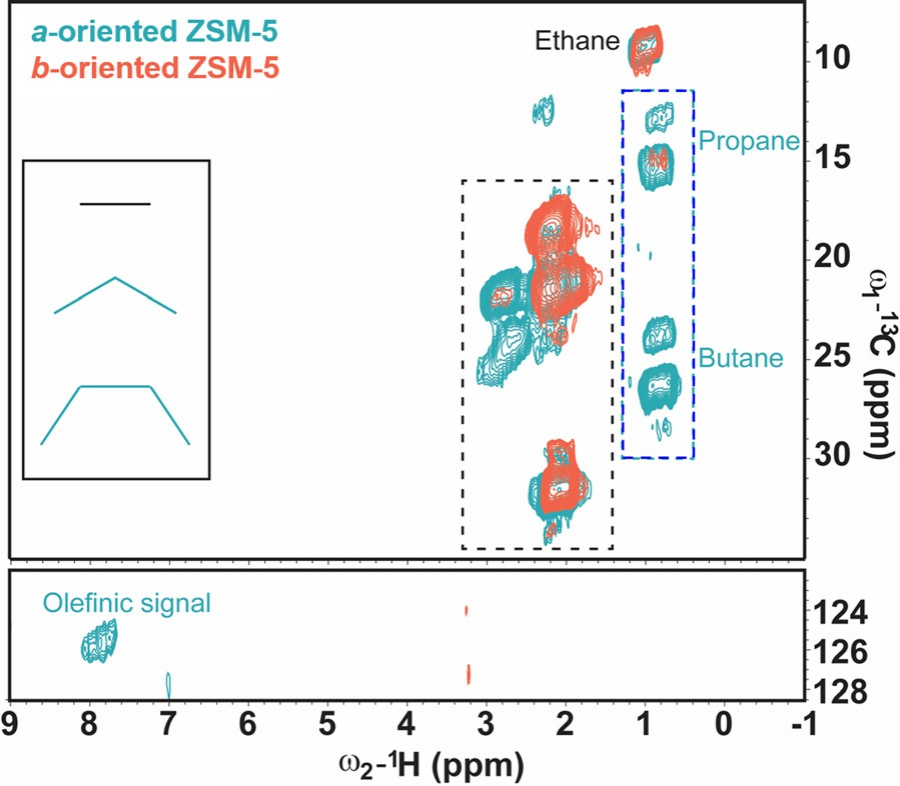
2D scalar‐based ^13^C‐^1^H MAS ssNMR spectra of mobile molecules trapped in the *a*‐oriented (blue) and *b*‐oriented (orange) zeolite crystals after 2 min of MTH process at 623 K. The spectra were recorded at 290 K using 16 kHz MAS. The black box indicates the methyl resonances corresponding to the species shown in the right panel of Figure 2. The blue box highlights the hydrogen‐transferred species only present in the *a*‐oriented zeolite ZSM‐5 crystals.^[60]^

2D ssNMR experiments were performed to elucidate in more detail the molecular signatures of the zeolite‐trapped hydrocarbon species. Identification of the rigid and mobile motifs of methanol molecules (see details in Figures S9 and S10 as well as corresponding discussion) adsorbed in the anisotropic crystals demonstrates the more constrained nature of the sinusoidal channels in the *a*‐orientation, compared to the straight channels in the *b*‐oriented counterpart. The rigid molecules were examined by 2D ^13^C‐^13^C ssNMR, as shown in Figure 4.^[56]^ Clear correlations between methyl groups at 20–25 ppm (^13^C) and aromatic at 130–140 ppm (^13^C) moieties revealed the presence of several HCP‐type intermediates.^[33]^ For the *a*‐oriented zeolite crystals (Figure 4 a), only one methyl resonance at 21.1 (^13^C) ppm was detected, while two interacting methyl resonances at 18.4 and 21.7 (^13^C) ppm were correlated with aromatic signals for the *b*‐oriented zeolite crystals (Figure 4 b). This suggests the formation of only symmetric methylated aromatics within the *a*‐oriented crystals, while asymmetric methylated aromatics were formed within the *b*‐oriented analog. This finding is confirmed by further experiments with longer ^13^C‐^13^C mixing time (Figure S11). Moreover, the 2D CP‐based ^13^C‐^1^H spectra (right panels, Figures 4 a and b) showed correlations between aliphatic at ≈2 (^1^H) ppm and aromatic at 7–8 (^1^H) ppm protons for the *a*‐oriented zeolite crystals, while they are absent in the *b*‐oriented zeolite crystals. This result demonstrates a higher degree of alkylation on the aromatics/olefinic HCP species in the *b*‐oriented zeolite crystals, which does not allow such ^13^C‐^1^H correlation as the aromatic protons are substituted by methyl groups. A further evidence is given by the observation of a higher ratio of ≈136 to ≈130 ppm species for the *b*‐oriented zeolite crystals, compared to the *a*‐oriented counterpart (Figure S12). In fact, the substitution of the aromatic proton by a methyl group causes a shift of the connected aromatic carbon to higher values, therefore moving the signal to 136 ppm. The higher degree of alkylation reveals the promotion of the aromatic cycle in the straight zeolite channels. It should be noted that the high ratio of ≈136 to ≈130 ppm signals could also be attributed to the fast formation of H‐deficient coke deposits, for example, naphthalene and anthracene, within the straight zeolite channels.[^38^, ^57^] Additionally, fewer resonances and a weaker signal intensity are found in all the proton‐detected ^13^C‐^1^H correlation spectra (Figures 4, 5 and S13–S15) for the *b*‐oriented zeolite ZSM‐5 crystal compared to the *a*‐oriented analogue, demonstrating the preferential formation of H‐deficient hydrocarbons in the *b*‐oriented zeolite crystals. Collectively, as summarized in Figure 4 c, it can be concluded that the straight zeolite channels favor the aromatic cycle due to the fast formation of internal coke deposits,[^38^, ^57^] with the production of a high degree of alkylated aromatics and asymmetric molecules, for example, 1,2,4‐trimethylbenzene. The latter are believed to be responsible for ethylene production.^[58]^ Therefore, more ethylene will be produced in the straight channels of zeolite ZSM‐5. Previously, we showed that the introduction of alkaline‐earth metals could dramatically suppress the aromatic cycles.[^35^, ^36^, ^59^] We speculate that these promotor species might be primarily located within the straight zeolite channels.

Next, we probed mobile molecules trapped in zeolites by 2D ssNMR measurements using through‐bond magnetization transfer schemes (Figure S4). In these ssNMR spectra (Figures 5, S13 and S14) certain resonances appear to be broadened or some signals show more than one peak for the same species, clearly indicating that the same molecule exists in different molecular environments inside the zeolite framework.^[33]^ As shown in Figures 5 and S13, the scalar‐based ^13^C‐^1^H correlation spectra identified ethane at 9.15 ppm (^13^C) and 0.98 ppm (^1^H) as well as propane at 18.9 ppm (^13^C) and 0.7 ppm (^1^H) in the *a*‐oriented zeolite crystals, while only ethane (8.97 (^13^C) and 0.99 (^1^H) ppm) was observed in the *b*‐oriented analog. Moreover, the resonances at ≈24 and 26 ppm (^13^C) and 0.9 ppm (^1^H) are only visible in the *a*‐oriented crystals and are compatible with butane and/or tetramethylethane signals (Figure S13). Based on the literature and by combining the spectra of the scalar‐based ^13^C‐^1^H and ^13^C‐^13^C correlations experiments, the signals were tentatively assigned to butane zeolite crystals. Taken together, these findings demonstrate the homologation reactions in the *a*‐oriented zeolite crystals, and thereby the preference of the olefin cycle in the sinusoidal zeolite channels.[^33^, ^61^]

### Spatial Distribution of Hydrocarbons in the Zeolites

**Figure 6:**
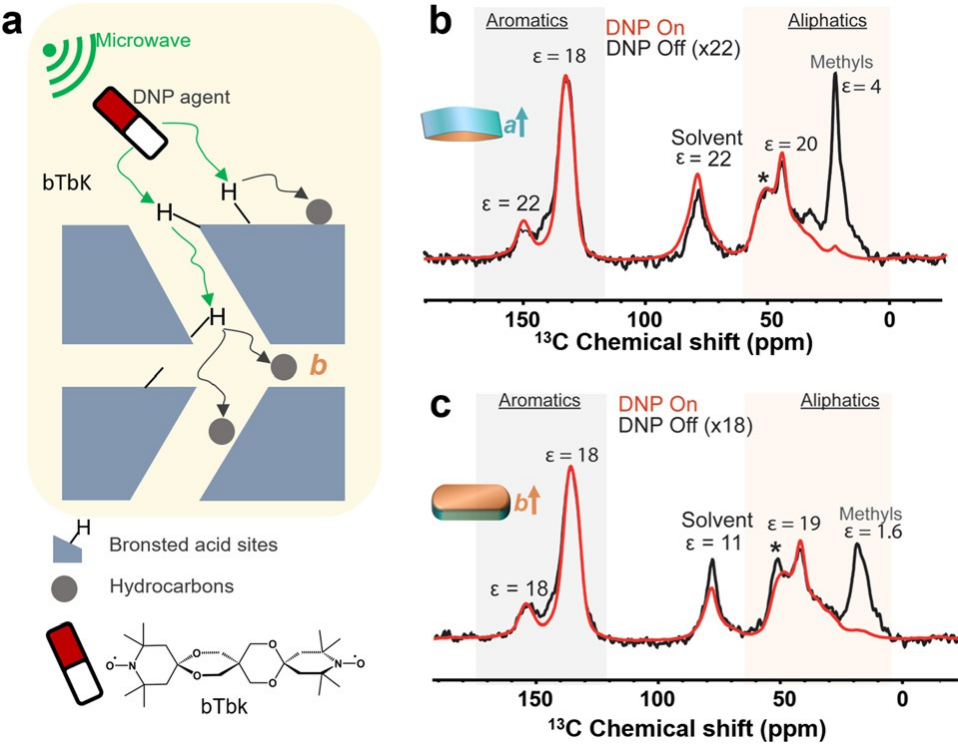
a) Simplified illustration of the hyperpolarization of carbon atoms of the hydrocarbons (in dark grey spheres) using DNP ssNMR spectroscopy. The light‐yellow background represents the solvent matrix with the DNP agent (red‐white ellipsoids) within the post‐impregnated zeolite. As a result of the microwave (μW) irradiation, electron polarization is transferred from the biradical (bTbK) to the zeolitic Brønsted acid sites and protons in the solvent. Next, ^1^H‐^1^H spin‐diffusion (SD) is allowed to occur at cryogenic temperatures, and then CP is used to transfer enhanced polarization to the dilute spins of the medium (i.e., zeolite‐trapped hydrocarbons in the present case). ^1^H‐^13^C CP DNP ssNMR spectra measured with the 400 MHz DNP system at 100 K for the b) *a*‐oriented and c) *b*‐oriented zeolite crystals after 2 min of the MTH process at 623 K. Black and red lines represent the spectra without and with irradiation of microwaves, respectively. MAS sidebands are indicated by *. The numbers denote the DNP‐enhancements computed from the intensity ratios of the spectra from on‐ and off‐DNP experiments.

Finally, the spatial distribution of hydrocarbons formed in the anisotropic crystals was examined using DNP experiments (Figure 6 a).^[62]^ DNP is a powerful technique for probing catalytic sites and active species/intermediates at the surface and in the pores of zeolites. In a typical DNP experiment, the nuclear signal is enhanced by microwave induced polarization transfer from unpaired electrons to nuclei (usually protons). For DNP, the porous samples are impregnated with a solution of stable radicals as electron source.[^43^, ^63^] These DNP agents are in close proximity to the material surface, enhancing proton polarization that can be transferred to the heteronuclei (e.g., ^13^C, ^27^Al, ^29^Si, ^119^Sn).[^62^, ^64^] In this work, samples for DNP experiments were prepared by impregnating the zeolites with a 16 mM solution of bis‐TEMPO‐bis‐ketal (bTbK) in tetrachloroethane (TCE). The bTbK radical is too large (ca. 6×13 Å and similar in size to benzanthracene), to diffuse into 10‐membered ring channels (ca. 5.3×5.6 Å), which will be filled with TCE and/or water from ambient moisture. The ^13^C DNP spectra (Figures 6 b and c) show significant enhancements for aromatic and methylene species. The negligible enhancement for methyl species is due to their fast rotational dynamics even at cryogenic temperatures, which provides an efficient relaxation sink under typical DNP MAS experimental conditions.^[65]^ For the aromatics at ≈150 ppm, DNP enhancements of 22 and 18 were observed for the *a*‐oriented and *b*‐oriented zeolite crystals, respectively. This indicates the formation of a greater fraction of the large aromatics at the sinusoidal channel dominated surface of the *a*‐oriented zeolite. Furthermore, a twofold enhancement of the solvent was observed for the *a*‐oriented zeolite crystals, compared to the *b*‐oriented analogue. As the DNP enhancement is largely confined to the surface, the above result suggests that the solvent remains more confined at the surface of the *a*‐oriented zeolite particles, while in the *b*‐oriented crystals it can more easily penetrate into the pores.^[62]^ This difference can be explained by a higher formation of aromatic species at the surface of the sinusoidal channel dominated particles, with respect to the *b*‐oriented crystal where the surface is dominated by the straight channels.

## Conclusion

In this work, detailed insights into the effect of channel geometry on the reaction intermediates of the MTH process within a single zeolite framework, that is, MFI, were obtained using a combination of anisotropic zeolite crystals and multi‐dimensional ssNMR spectroscopy. The results unequivocally show that subtle differences in the zeolite channel geometry dramatically alter the configuration of reaction intermediates. In particular, the more constrained sinusoidal channels in the *a*‐oriented zeolite ZSM‐5 crystals favor the olefin cycle with the promotion of homologation of alkanes. The more extended straight channels in the *b*‐oriented catalysts facilitate the aromatic cycle, as given by the higher degree of alkylation, and are therefore responsible for producing ethylene.^[38]^ This study further promotes the concept of the hybrid inorganic‐organic nature of an active MTH catalyst, where the distinct host‐guest chemistry between the shape and topology of the internal zeolite pore structure and the trapped hydrocarbons plays an important role in control of the ultimate product selectivity. Such knowledge will not only be useful for the development of superior and/or improved catalysts for the MTH reaction (by designing zeolite‐based catalysts to mimic the transition states of the reactions catalyzed),^[39]^ but will also contribute to our understanding of the host‐guest chemistry and shape selectivity.

## Conflict of interest

The authors declare no conflict of interest.

## Supporting information

As a service to our authors and readers, this journal provides supporting information supplied by the authors. Such materials are peer reviewed and may be re‐organized for online delivery, but are not copy‐edited or typeset. Technical support issues arising from supporting information (other than missing files) should be addressed to the authors.

Supplementary

Supplementary

Supplementary

## References

[1] A. Corma , J. Catal. 2003, 216, 298–312.

[2] B. Smit , T. L. M. Maesen , Nature 2008, 451, 671–678. 18256663 10.1038/nature06552

[3] S. M. Csicsery , Zeolites 1984, 4, 202–213.

[4] U. Olsbye , S. Svelle , M. Bjørgen , P. Beato , T. V. W. Janssens , F. Joensen , S. Bordiga , K. P. Lillerud , Angew. Chem. Int. Ed. 2012, 51, 5810–5831; 10.1002/anie.201103657 22511469

[5] P. Tian , Y. Wei , M. Ye , Z. Liu , ACS Catal. 2015, 5, 1922–1938.

[6] M. Stöcker , Microporous Mesoporous Mater. 1999, 29, 3–48.

[7] I. Yarulina , A. D. Chowdhury , F. Meirer , B. M. Weckhuysen , J. Gascon , Nat. Catal. 2018, 1, 398–411.

[8] S. Teketel , W. Skistad , S. Benard , U. Olsbye , K. P. Lillerud , P. Beato , S. Svelle , ACS Catal. 2012, 2, 26–37.

[9] S. Teketel , U. Olsbye , K.-P. Lillerud , P. Beato , S. Svelle , Microporous Mesoporous Mater. 2010, 136, 33–41.

[10] Y. Chu , X. Sun , X. Yi , L. Ding , A. Zheng , F. Deng , Catal. Sci. Technol. 2015, 5, 3507–3517.

[11] Z. Liu , Y. Chu , X. Tang , L. Huang , G. Li , X. Yi , A. Zheng , J. Phys. Chem. C 2017, 121, 22872–22882.

[12] M. Bjørgen , F. Joensen , K.-P. Lillerud , U. Olsbye , S. Svelle , Catal. Today 2009, 142, 90–97.

[13] J. Li , Y. Wei , J. Chen , S. Xu , P. Tian , X. Yang , B. Li , J. Wang , Z. Liu , ACS Catal. 2015, 5, 661–665.

[14] J. Chen , J. Li , Y. Wei , C. Yuan , B. Li , S. Xu , Y. Zhou , J. Wang , M. Zhang , Z. Liu , Catal. Commun. 2014, 46, 36–40.

[15] I. Pinilla-Herrero , U. Olsbye , C. Márquez-Álvarez , E. Sastre , J. Catal. 2017, 352, 191–207.

[16] I. Pinilla-Herrero , C. Márquez-Álvarez , E. Sastre , Catal. Sci. Technol. 2017, 7, 3892–3901.

[17] J. H. Kang , F. H. Alshafei , S. I. Zones , M. E. Davis , ACS Catal. 2019, 9, 6012–6019.

[18] P. Ferri , C. Li , C. Paris , A. Vidal-Moya , M. Moliner , M. Boronat , A. Corma , ACS Catal. 2019, 9, 11542–11551.

[19] Y. Bhawe , M. Moliner-Marin , J. D. Lunn , Y. Liu , A. Malek , M. E. Davis , ACS Catal. 2012, 2, 2490–2495.

[20] M. Zhang , S. Xu , Y. Wei , J. Li , J. Chen , J. Wang , W. Zhang , S. Gao , X. Li , C. Wang , Z. Liu , RSC Adv. 2016, 6, 95855–95864.

[21] N. Wang , Y. Zhi , Y. Wei , W. Zhang , Z. Liu , J. Huang , T. Sun , S. Xu , S. Lin , Y. He , A. Zheng , Z. Liu , Nat. Commun. 2020, 11, 1079. 32103001 10.1038/s41467-020-14493-9 PMC7044299

[22] W. Zhang , J. Chen , S. Xu , Y. Chu , Y. Wei , Y. Zhi , J. Huang , A. Zheng , X. Wu , X. Meng , F. Xiao , F. Deng , Z. Liu , ACS Catal. 2018, 8, 10950–10963.

[23] S. Teketel , S. Svelle , K.-P. Lillerud , U. Olsbye , ChemCatChem 2009, 1, 78–81.

[24] J. F. Haw , W. Song , D. M. Marcus , J. B. Nicholas , Acc. Chem. Res. 2003, 36, 317–326. 12755641 10.1021/ar020006o

[25] S. Svelle , F. Joensen , J. Nerlov , U. Olsbye , K.-P. Lillerud , S. Kolboe , M. Bjørgen , J. Am. Chem. Soc. 2006, 128, 14770–14771. 17105263 10.1021/ja065810a

[26] S. Ilias , A. Bhan , ACS Catal. 2013, 3, 18–31.

[27] U. Olsbye , S. Svelle , K. P. Lillerud , Z. H. Wei , Y. Y. Chen , J. F. Li , J. G. Wang , W. B. Fan , Chem. Soc. Rev. 2015, 44, 7155–7176. 26185806 10.1039/c5cs00304k

[28] W. Song , H. Fu , J. F. Haw , J. Am. Chem. Soc. 2001, 123, 4749–4754. 11457284 10.1021/ja0041167

[29] J. F. Haw , D. M. Marcus , Top. Catal. 2005, 34, 41–48.

[30] C. Wang , Q. Wang , J. Xu , G. Qi , P. Gao , W. Wang , Y. Zou , N. Feng , X. Liu , F. Deng , Angew. Chem. Int. Ed. 2016, 55, 2507–2511; 10.1002/anie.201510920 26732748

[31] C. Wang , J. Xu , Q. Wang , X. Zhou , G. Qi , N. Feng , X. Liu , X. Meng , F. Xiao , F. Deng , ACS Catal. 2017, 7, 6094–6103.

[32] A. D. Chowdhury , K. Houben , G. T. Whiting , M. Mokhtar , A. M. Asiri , S. A. Al-Thabaiti , S. N. Basahel , M. Baldus , B. M. Weckhuysen , Angew. Chem. Int. Ed. 2016, 55, 15840–15845; 10.1002/anie.201608643 PMC5214583 27805783

[33] A. D. Chowdhury , A. Lucini Paioni , K. Houben , G. T. Whiting , M. Baldus , B. M. Weckhuysen , Angew. Chem. Int. Ed. 2018, 57, 8095–8099; 10.1002/anie.201803279 PMC6563700 29710435

[34] A. D. Chowdhury , A. Lucini Paioni , G. T. Whiting , D. Fu , M. Baldus , B. M. Weckhuysen , Angew. Chem. Int. Ed. 2019, 58, 3908–3912; 10.1002/anie.201814268 PMC6519145 30681254

[35] S. Bailleul , I. Yarulina , A. Hoffman , A. Dokania , E. Abou-Hamad , A. Dutta Chowdhury , G. Pieters , J. Hajek , K. De Wispelaere , M. Waroquier , J. Gascon , V. Van Speybroeck , J. Am. Chem. Soc. 2019, 141, 14823–14842. 31464134 10.1021/jacs.9b07484 PMC6753656

[36] A. Dutta Chowdhury , I. Yarulina , E. Abou-Hamad , A. Gurinov , J. Gascon , Chem. Sci. 2019, 10, 8946–8954. 32190235 10.1039/c9sc02215e PMC7068724

[37] P. Cnudde , R. Demuynck , S. Vandenbrande , M. Waroquier , G. Sastre , V. V. Speybroeck , J. Am. Chem. Soc. 2020, 142, 6007–6017. 32157875 10.1021/jacs.9b10249

[38] D. Fu , O. van der Heijden , K. Stanciakova , J. E. Schmidt , B. M. Weckhuysen , Angew. Chem. Int. Ed. 2020, 59, 15502–15506; 10.1002/anie.201916596 PMC7496746 32026555

[39] E. M. Gallego , M. T. Portilla , C. Paris , A. León-Escamilla , M. Boronat , M. Moliner , A. Corma , Science 2017, 355, 1051–1054. 28280200 10.1126/science.aal0121

[40] C. Li , C. Paris , J. Martínez-Triguero , M. Boronat , M. Moliner , A. Corma , Nat. Catal. 2018, 1, 547–554.

[41] A. A. Labokha , S. Gradmann , S. Frey , B. B. Hülsmann , H. Urlaub , M. Baldus , D. Görlich , EMBO J. 2013, 32, 204–218. 23202855 10.1038/emboj.2012.302 PMC3553378

[42] A. Lesage , M. Lelli , D. Gajan , M. A. Caporini , V. Vitzthum , P. Miéville , J. Alauzun , A. Roussey , C. Thieuleux , A. Mehdi , G. Bodenhausen , C. Copéret , L. Emsley , J. Am. Chem. Soc. 2010, 132, 15459–15461. 20831165 10.1021/ja104771z

[43] Q. Z. Ni , E. Daviso , T. V. Can , E. Markhasin , S. K. Jawla , T. M. Swager , R. J. Temkin , J. Herzfeld , R. G. Griffin , Acc. Chem. Res. 2013, 46, 1933–1941. 23597038 10.1021/ar300348n PMC3778063

[44] A. J. Rossini , A. Zagdoun , M. Lelli , A. Lesage , C. Copéret , L. Emsley , Acc. Chem. Res. 2013, 46, 1942–1951. 23517009 10.1021/ar300322x

[45] P. C. A. van der Wel , K.-N. Hu , J. Lewandowski , R. G. Griffin , J. Am. Chem. Soc. 2006, 128, 10840–10846. 16910679 10.1021/ja0626685

[46] D. Xiao , S. Xu , N. J. Brownbill , S. Paul , L.-H. Chen , S. Pawsey , F. Aussenac , B.-L. Su , X. Han , X. Bao , Z. Liu , F. Blanc , Chem. Sci. 2018, 9, 8184–8193. 30568769 10.1039/c8sc03848a PMC6254210

[47] J. Martinez-Ortigosa , J. Simancas , J. A. Vidal-Moya , P. Gaveau , F. Rey , B. Alonso , T. Blasco , J. Phys. Chem. C 2019, 123, 22324–22334.

[48] D. Mores , J. Kornatowski , U. Olsbye , B. M. Weckhuysen , Chem. Eur. J. 2011, 17, 2874–2884. 21305622 10.1002/chem.201002624

[49] M. J. Wulfers , F. C. Jentoft , ACS Catal. 2014, 4, 3521–3532.

[50] F. Deng , C. Wang , J. Xu , ChemCatChem 2019, 12, 965–980.

[51] G. A. Morris , R. Freeman , J. Am. Chem. Soc. 1979, 101, 760–762.

[52] A. Pines , M. G. Gibby , J. S. Waugh , J. Chem. Phys. 1973, 59, 569–590.

[53] O. C. Andronesi , S. Becker , K. Seidel , H. Heise , H. S. Young , M. Baldus , J. Am. Chem. Soc. 2005, 127, 12965–12974. 16159291 10.1021/ja0530164

[54] J. F. Haw , J. B. Nicholas , W. Song , F. Deng , Z. Wang , T. Xu , C. S. Heneghan , J. Am. Chem. Soc. 2000, 122, 4763–4775.

[55] C. Wang , Y. Chu , A. Zheng , J. Xu , Q. Wang , P. Gao , G. Qi , Y. Gong , F. Deng , Chem. Eur. J. 2014, 20, 12432–12443. 25178472 10.1002/chem.201403972

[56] M. Weingarth , D. E. Demco , G. Bodenhausen , P. Tekely , Chem. Phys. Lett. 2009, 469, 342–348.

[57] D. Mores , E. Stavitski , M. H. F. Kox , J. Kornatowski , U. Olsbye , B. M. Weckhuysen , Chem. Eur. J. 2008, 14, 11320–11327. 19021162 10.1002/chem.200801293

[58] F. L. Bleken , T. V. W. Janssens , S. Svelle , U. Olsbye , Microporous Mesoporous Mater. 2012, 164, 190–198.

[59] I. Yarulina , K. D. Wispelaere , S. Bailleul , J. Goetze , M. Radersma , E. Abou-Hamad , I. Vollmer , M. Goesten , B. Mezari , E. J. M. Hensen , J. S. Martínez-Espín , M. Morten , S. Mitchell , J. Perez-Ramirez , U. Olsbye , B. M. Weckhuysen , V. van Speybroeck , F. Kapteijn , J. Gascon , Nat. Chem. 2018, 10, 804–812. 29941905 10.1038/s41557-018-0081-0

[60] S. Müller , Y. Liu , F. M. Kirchberger , M. Tonigold , M. Sanchez-Sanchez , J. A. Lercher , J. Am. Chem. Soc. 2016, 138, 15994–16003. 27960343 10.1021/jacs.6b09605

[61] X. Sun , S. Mueller , Y. Liu , H. Shi , G. L. Haller , M. Sanchez-Sanchez , A. C. van Veen , J. A. Lercher , J. Catal. 2014, 317, 185–197.

[62] D. Mance , J. van der Zwan , M. E. Z. Velthoen , F. Meirer , B. M. Weckhuysen , M. Baldus , E. T. C. Vogt , Chem. Commun. 2017, 53, 3933–3936. 10.1039/c7cc00849j 28327736

[63] T. Maly , G. T. Debelouchina , V. S. Bajaj , K.-N. Hu , C.-G. Joo , M. L. Mak-Jurkauskas , J. R. Sirigiri , P. C. A. van der Wel , J. Herzfeld , R. J. Temkin , R. G. Griffin , J. Chem. Phys. 2008, 128, 052211. 18266416 10.1063/1.2833582 PMC2770872

[64] P. Wolf , M. Valla , A. J. Rossini , A. Comas-Vives , F. Núñez-Zarur , B. Malaman , A. Lesage , L. Emsley , C. Copéret , I. Hermans , Angew. Chem. Int. Ed. 2014, 53, 10179–10183; 10.1002/anie.201403905 25079352

[65] A. J. Rossini , A. Zagdoun , F. Hegner , M. Schwarzwälder , D. Gajan , C. Copéret , A. Lesage , L. Emsley , J. Am. Chem. Soc. 2012, 134, 16899–16908. 22967206 10.1021/ja308135r

