# Supplementary material for: Elucidating Zeolite Channel Geometry–Reaction Intermediate Relationships for the Methanol‐to‐Hydrocarbon Process
Source: Angew Chem Int Ed Engl. 2020 Sep 11;59(45):20024–30. doi: 10.1002/anie.202009139 (PMC7692936; doi:10.1002/anie.202009139)
Supplement: Supplementary file 2 — Supplementary [file ANIE-59-20024-s002.pdf]

Angewandte Chemie
Eine Zeitschrift der Gesellschaft Deutscher Chemiker

Supporting Information

Elucidating Zeolite Channel Geometry–Reaction Intermediate Relationships for the Methanol-to-Hydrocarbon Process

Donglong Fu[+], Alessandra Lucini Paioni[+], Cheng Lian, Onno van der Heijden, Marc Baldus, and Bert M. Weckhuysen**

anie_202009139_sm_miscellaneous_information.pdf
anie_202009139_sm_3-SI-movieS1.mov

S1. Chemicals and Materials 2

S2. Methanol-to-Hydrocarbons Reactions 3

S3. Solid-State Nuclear Magnetic Resonance Spectroscopy 3

S4. Molecular Dynamics Simulation 4

S5. Supplementary Figures S4-S15 and Tables S3-S5 7

S6. References 20

S1. Chemicals and Materials

Chemicals. Tetraethyl orthosilicate (TEOS, 98% (GC), Sigma Aldrich), tetrapropylammonium hydroxide (TPAOH, 1M aq., Alfa Aesar), bis(hexamethylene)triamine, 1-iodepropane (99%, Sigma-Aldrich), 2-butanone (>99%m, Sigma-Aldrich), anhydrous potassium carbonate (≥99.0% (T), anhydrous, BioUltra, Sigma-Aldrich), diethyl ether (ACS reagent, , ≥99.0%, anhydrous, ACS reagent, Sigma-Aldrich), ethyl acetate (99.8%, anhydrous, Sigma-Aldrich), potassium hydroxide (KOH, pellets, 85%, Alfa Aesar), aluminium sulphate hexadecahydrate ($Al_2(SO_4)_3{\cdot}18H_2O$, 98%, Sigma-Aldrich), ammonium nitrate (NH_4NO_3, >99%, Acros Organics), sodium aluminate (Al_2O_3: 50-56%, Na_2O: 30-37%, Sigma-Aldrich), ethanol (anhydrous, absolute, Biosolve), ^{13}C-methanol (99 atom% ^{13}C, Cambridge Isotope Laboratories), sulfuric acid (95%, reagent grade, Fischer scientific), bis-TEMPO-bis-ketal (bTbk, homemade biradical for DNP experiments), 1,1,2,2-tetrachloroethane (TCE, ≥98.0%, Sigma-Aldrich) were used as received.

Materials synthesis. The *a*-oriented and *b*-oriented zeolite ZSM-5 crystals were prepared with the method adjusted from literatures using trimer-tetrapropylammonium cation (trimer-TPA$^+$) and tetrapropylammonium cation (TPA$^+$) as the structure-directing agent (SDA, Figure S1), respectively.[1–3] The chemical compositions of the solutions were adjusted to obtain anisotropic zeolite ZSM-5 crystals with comparable framework Si/Al ratios. Typically, the synthesis solution of *a*-oriented zeolite ZSM-5 crystals was prepared by introducing TEOS into the solution containing trimer-TPA^{3+}, KOH and H_2O, as described in literature [4]. Then required amount of aluminum sulphate was added to the clear mixture (pH≈13) obtained after stirring in a sealed liner for 4 h at room temperature. The final solution with a composition of 6 TEOS:0.066 Al:0.75 Trimer-TPA^{3+}:1.17 KOH:620 H_2O was charged into a Teflon liner after stirring at room temperature for an extra hour. The liner was placed in an autoclave and heated to synthesis temperature (448 K) in a rotation oven for 24 h. Preparation of the *b*-oriented zeolite ZSM-5 crystals was adapted from the reported method.[2] In a typical synthesis, sodium aluminate was added to a mixture of 0.445 g NaOH solution (10 wt% of solution) and 1.24 g milliQ H_2O. TPAOH was added to this solution followed by TEOS. The final solution with a composition of 6 TEOS:0.066 Al:0.9 TPAOH:620 H_2O was obtained after 2 h stirring at room temperature in a sealed bottle to allow for the hydrolysis of TEOS. The solution was transferred into 20 mL Teflon lined stainless steel autoclaves, and the hydrothermal reaction was carried out under rotation oven at 443 K for 24 h. The H-form catalysts were obtained by calcination of ion exchanged crystals. Typically, ~200 mg catalyst and ~30 mL 1 M aqueous ammonium nitrate were mixed and stirred overnight at ~333 K. The crystals were washed again using only water, and dried overnight at 353 K.

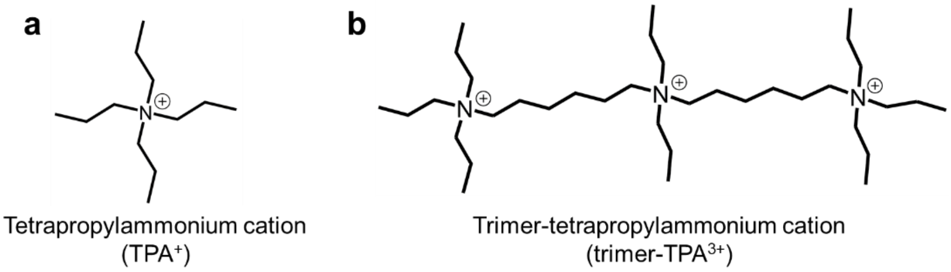

Figure S1. Molecular structures of the employed structure-directing agents (SDAs). a) Tetrapropylammonium cation (TPA+) and b) trimer-tetropropylammonium (trimer-TPA^{3+}) for the synthesis of *b*-oriented and *a*-oriented zeolite ZSM-5 crystals, respectively.

Materials characterization. The crystallinity and morphology of the as-prepared anisotropic zeolite ZSM-5 crystals were measured using X-ray diffraction (XRD) and scanning electron microscope (SEM), respectively. The XRD patterns were collected using a Bruker D2 Phaser (2nd Gen) instrument using a cobalt radiation source, Co k_α = 1.789 Å. The samples were rotated at 15 rad/min. The SEM images of the zeolite ZSM-5 crystals were examined using an XL-30 (Philips) microscope operating at an accelerating voltage of 5 kV. Before measurement, the surface of the anisotropic zeolite crystals was coated with *ca*. 5 nm thickness of Pt to avoid charging effects. The framework Si/Al ratios of the

anisotropic zeolite ZSM-5 crystals were examined using magic angle spinning (MAS) solid-state nuclear magnetic resonance (ssNMR, see details in section S3) spectroscopy.

S2. Methanol-to-Hydrocarbons Reactions

The methanol-hydrocarbons (MTH) reactions of the anisotropic zeolite ZSM-5 crystals were performed at 623 K in a quartz, rectangular fixed-bed reactor (ID= 6 mm× 3 mm). *In situ* UV/Vis diffuse reflectance spectroscopy (DRS) was applied simultaneously to monitor the entrapped hydrocarbon pool (HCP) species during the MTH.[4,5] *In situ* UV/Vis DRS spectra were obtained using an AvaSpec 2048L spectrometer connected to a high-temperature UV/Vis optical fiber probe, which was used to collect spectra in reflection mode. The measurements were performed in the wavelength range of 200-1000 nm (11000-50000 cm^{-1} in wavenumber). Typically, *ca.* 100 mg of catalysts was loaded and calcined under a flow of pure O_2 with a ramp of 15 K/min to 823 K and hold for 120 min. The MTH reaction was then performed after first cooling the catalysts to 623 K and switching to He atmosphere. A weight hourly space velocity (WHSV) of 5 h^{-1} was obtained with a ^{13}C-methanol saturation of *ca.* 14.5 % by flowing the carrier gas through a saturator containing ^{13}C-methanol at 293 K. After 2 min of reaction the flow was switched back to He, and the heating furnace was removed. The reaction was quenched by rapid cooling of the reactor using compressed air.

S3. Solid-State Nuclear Magnetic Resonance Spectroscopy

All solid-state nuclear magnetic resonance (ssNMR) experiments were performed at room temperature (298 K) and at an MAS frequency of 16 kHz on a Bruker 500MHz wide-bore magnet with an AVANCE-III console and equipped with a 3.2 mm HXY probe in double channel mode. For the ^{27}Al NMR experiments, a radio frequency (RF) field of 50 kHz was used for the π/12 pulse to excite only the central transition, followed by an acquisition time of 4.5 ms. 10240 scans were accumulated using an inter-scan delay of 1 s. The ^{27}Al chemical shift was externally referenced to an aluminium nitrate solution ($Al(NO_3)_3$(aq)). The 1D NMR spectra were processed using a line-broadening of 100 Hz. ^{29}Si NMR experiments were performed with a MAS frequency of 12 kHz and referencing was done externally to tetramethylsilane (TMS). ^{29}Si NMR spectra were recorded using direct excitation (DE). A RF field of 54 kHz was used for the measurement of ^{29}Si DE spectra, acquired with 1024 scans and an inter-scan delay of 10 s. A line broadening of 50 Hz was applied for spectral processing. All spectra were processed and analyzed with Bruker Topspin 3.5. The silicon to aluminum (Si/Al) ratio was estimated using Lowenstein's rule and using *eq. S1*, where $I_{Si(nAl)}$ is the intensity of the individual Si(*n*Al) peaks (% area) obtained from the corresponding deconvoluted spectra and n_{max} = 1 in the present case.[6]

$$Si/Al(framework) = \frac{\sum_{n=0}^{4} I_{Si(nAl)}}{\sum_{n=0}^{4} 0.25n * I_{Si(nAl)}} \quad \text{(Eq. } S1\text{)}$$

The ^{1}H and ^{13}C NMR experiments were performed at room temperature (298 K) and at an MAS frequency of 16 kHz. Note that effective sample temperatures can be 5-10 degrees higher due to frictional heating. Referencing of ^{1}H and ^{13}C chemical shifts was done externally to adamantane. Hard ^{1}H and ^{13}C pulses were applied with field strength of 88 kHz and 45 kHz, respectively. SPINAL64 ^{1}H decoupling (at a field strength of 88 KHz) was applied during acquisition for the 1D and 2D ^{13}C detected spectra.[7] *1D ssNMR*: The 1D ^{1}H-^{13}C cross-polarization (CP) spectrum was recorded using a 1.5 s recycle delay, 12 ms acquisition time and an accumulation of 4096 scans.[8] CP was achieved using a 38 kHz ^{13}C field and 78 kHz 70-100% ramped ^{1}H field and a contact time of 0.7 ms. The 1D ^{13}C DE spectrum was recorded using a 2 s recycle delay, 14 ms acquisition time and an accumulation of 4096 scans. The 1D ^{1}H-^{13}C insensitive nuclei enhanced by polarization transfer (INEPT) spectrum was recorded using a 1.5 s recycle delay, a 33 ms acquisition time and after accumulation of 4096 scans.[9] 1D CP, DE and INEPT spectra were processed using 150 Hz line-broadening.

2D ssNMR for probing rigid molecules. The dipolar based 2D ^{13}C-^{1}H correlation spectrum was obtained using a 0.7 ms ^{1}H-^{13}C CP period and a 0.4 ms ^{13}C-^{1}H CP period. Background signals from catalyst protons were suppressed by a 8 kHz MISSISSIPPI block (τ = 5 ms, N = 2) prior to the last CP.[10] ^{13}C PISSARRO (Phase-Inverted Supercycled Sequence for Attenuation of Rotary Resonance) decoupling was applied during acquisition.[11] The recycle delay was 1.5 s,

acquisition times 10 ms (F2, ^{1}H) and 6 ms (F1, ^{13}C) and 128 scans. The spectrum was processed using a 0.33 π shifted sine squared window function in both dimensions. 2D^{13}C-^{13}C spectra were recorded using a 1.5 s recycle delay, 10 ms (F2) and 3.5 ms (F1) acquisition time and an accumulation of 1024 scans. ^{13}C-^{13}C mixing was achieved through proton-driven spin-diffusion using phase-alternated-recoupling-irradiation-schemes (PARIS) for 30 ms.[12] Spectra were processed using a 0.33 π shifted sine squared window function in both dimensions.

2D ssNMR for probing mobile molecules. A 2D ^{13}C-^{1}H HSQC (Heteronuclear Single Quantum Coherence) spectrum was recorded using 155 Hz for J in the INEPT delay.[13] A 1.5 s recycle delay was used, acquisition times were 30 ms (F2, ^{1}H) and 4 ms (F1, ^{13}C) ms and 256 scans were accumulated. 6 kHz GARP ^{13}C decoupling was used during detection.[14] Prior to Fourier transformation, 0.5 π shifted sine squared function was applied in both dimensions. The 2D ^{1}H-^{13}C spectrum was recorded using a 1.5 s recycle delay, 15 ms (F2) or 4 ms (F1) acquisition time and an accumulation of 256 scans. A J coupling of 155 Hz was used to set the INEPT delay. A 10 kHz WALTZ ^{1}H decoupling was used during detection.[15] Prior to Fourier transformation, a window function corresponding to a 0.5 π shifted sine squared function was applied to both dimensions. The 2D ^{13}C-^{13}C TOBSY was recorded using a mixing time of 4 ms, a 2 s recycle delay and 16 ms (F2) or 3 ms (F1) acquisition time using 384 scans.[16] The spectrum was recorded using 155 Hz for J in the INEPT delay and processed using 200 Hz line-broadening in the F2 dimension and a 0.5 π shifted sine squared function to the F1 dimension. 10 kHz WALTZ ^{1}H decoupling was used during detection.

Dynamic Nuclear Polarization (DNP) MAS NMR. DNP experiments were conducted at low temperature (100 K) using 3.2 mm triple-resonance (^{1}H, ^{13}C, and ^{15}N) MAS probe heads in static magnetic fields of 9.4, corresponding to proton/electron resonance frequencies of 400 MHz/263 GHz (Bruker BioSpin). DNP samples were prepared by wetting, using a solution of 16 mM bTbk in tetrachloroethane.[17–19] The ^{1}H-^{13}C cross polarization DNP experiments presented in Figure 6 were recorded using a 2 s recycle delay, a 15 ms acquisition time and an accumulation of 1024 scans. A CP contact time of 0.6 ms was used and SPINAL-64 proton decoupling at a field strength of 83 kHz was applied during acquisition. The spectra were processed with 75 Hz line-broadening. All spectra were recorded at a MAS rate of 8 kHz. DNP enhancements were obtained by scaling the signal intensities of spectra measured under DNP with the corresponding spectrum obtained without microwave irradiation at 100K. Spinning sidebands were identified by varying MAS speeds. The DNP samples were prepared following the same procedure in both cases.[20]

*Analysis of ssNMR Data***.** All NMR spectra were processed with Bruker Topspin3.5 and analyzed using SPARKY.[21]

S4. Molecular Dynamics Simulation

Molecular dynamics (MD) simulation. The commercial software Materials Studio (Accelrys, Inc.) was used to calculate diffusivity of methanol molecules inside pure-Si zeolite ZSM-5 (MFI framework, Figure S2). The COMPASS force field was chosen to describe the interactions between atoms in the simulations. The diffusion coefficients (D) were evaluated from the mean squared displacement (MSD), according to the Einstein relation. Since the value of the MSD is already averaged over the number of atoms, the Einstein equation simplifies to $D = k/6$, where k is slope of MSD.

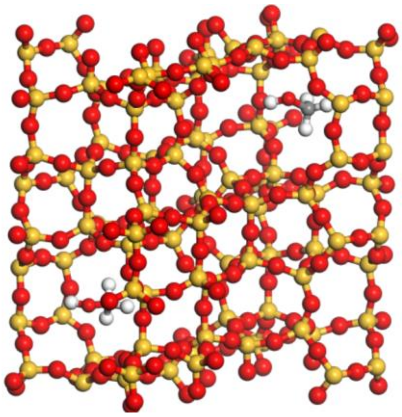

Figure S2. Molecular dynamics (MD) model for methanol molecules in a pure-Si zeolite ZSM-5.

Continuum model for the diffusion. A 2D model (Figure S3) was constructed for the diffusion of small molecules inside porous ZSM-5 by using COMSOL Multiphysics, the software of finite element method. The entire model covers 2150 nm by 642 nm, with a comparable to the aspect ratio of the anisotropic zeolite ZSM-5 crystals. Methanol molecule moves from right and top to left and bottom across the geometry. Since the channels are set as 40 nm in width, and the *D* is from the above MD simulation. The flow in the pores does not penetrate the solid grains. The inlet and outlet fluid pressures are known. The time-dependent concentration follows:

$$\frac{\partial c}{\partial t} + \nabla \cdot (-D\nabla c) = 0 \qquad \text{(Eq. } S2\text{)}$$

where c denotes concentration (mol/m^3 using SI units) and D the diffusion coefficient (m^2/s) of the small molecules.

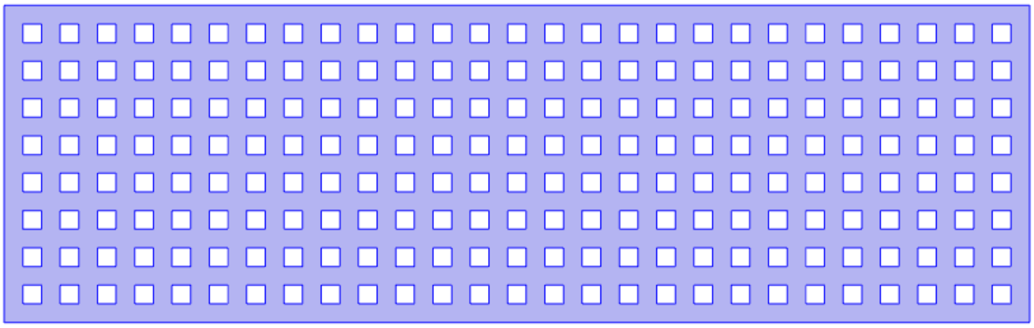

Figure S3. 2D porous structure model. The domain colored in blue is accessible for diffusion.

Stokes equation (*eq. S3*) was used to calculate the flow in the channels. The incompressible assumption together with the stationary condition reads:

$$0 = -\nabla p + \nabla \cdot \mu(\nabla\mu + \nabla\mu^{T}),\ \nabla \cdot \mu = 0 \qquad \text{(Eq. } S3\text{)}$$

Here, p is the pressure (SI unit: Pa), μ is the velocity field (SI unit: m/s), and μ is the dynamic viscosity of the fluid (SI unit: Pa·s). At the physical boundaries, the inlet pressure and the outlet pressure are known. Velocities are zero at the boundaries, which implies a no-slip condition. Tables S1 and S2 summarize the boundary conditions and the simulation results obtained from the model, respectively.

Table S1. Boundary conditions for the continuum diffusion model.

Boundary type	Boundary condition	Value
Inlet	Pressure, no viscous stress	p=p_0
Outlet	Pressure, no viscous stress	p=0

Note: p_0 is a specified pressure drop.

Table S2. Simulation results obtained from the continuum diffusion model.

Quantity	Value	Description
ρ_0	10 kg/m^3	Fluid density
μ_0	0.001 Pa*s	Fluid dynamic viscosity
p_0	1000 Pa	Pressure drop
D	1×10^{-13}m^2/s	Diffusion coefficient

S5. Supplementary Figures S4-S15 and Tables S3-S5

S5.1. Research methodology of this work

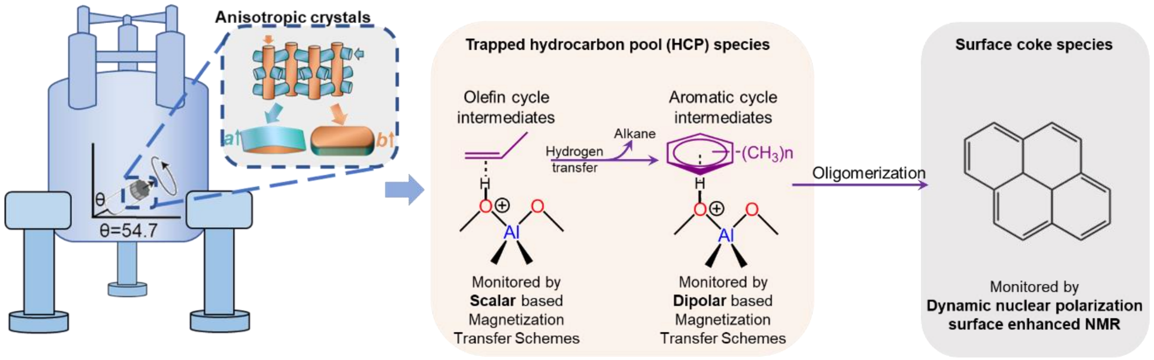

Figure S4. Using magic angle spinning (MAS) solid-state nuclear magnetic resonance spectroscopy (ssNMR), the reactive MTH intermediates formed in the different zeolite channels over the anisotropic zeolite ZSM-5 crystals were separated and identified on the basis of their mobility. The rigid and mobile hydrocarbons trapped within zeolites were spectrally separated using different magnetization transfer schemes that invoke through-space (dipolar transfer such as in cross-polarization, CP) or through-bond (scalar interactions such as in insensitive nuclei enhanced by polarization transfer, INEPT), respectively.[22,23] The spatial distribution of hydrocarbons formed in the anisotropic zeolite ZSM-5 crystals was identified using dynamic nuclear polarization (DNP) surface enhanced NMR spectroscopy.[18,19,24]

S5.2. Crystallographic details for the anisotropic zeolite ZSM-5 crystals

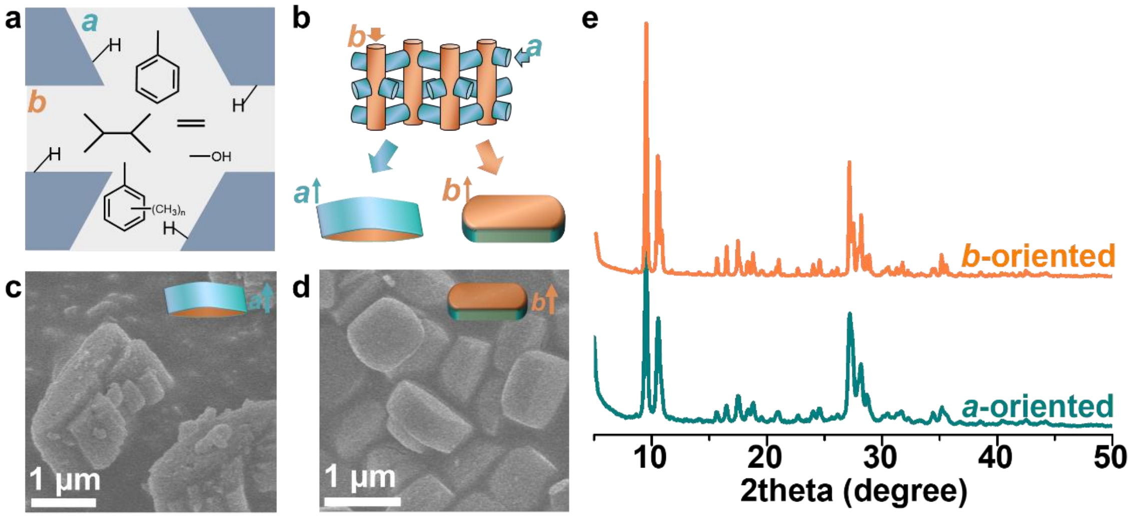

Figure S5. a,b) Schematic illustrations of a) the hydrocarbon pool (HCP) species trapped within a MFI framework (2D projection) during the methanol-to-hydrocarbons (MTH) and b) the morphology of the *a*-oriented and *b*-oriented zeolite ZSM-5 crystals. The surface of the *a*-oriented and *b*-oriented zeolite crystals are dominated by the sinusoidal channels (blue) and straight channels (orange), respectively. c,d) Scanning electron microscopy (SEM) images of the c) *a*-oriented and d) *b*-oriented zeolite ZSM-5 crystals. e) X-ray diffraction (XRD) patterns of the *a*-oriented (blue) and *b*-oriented (orange) zeolite ZSM-5 crystals.

S5.3. Solid-state NMR spectroscopy of the framework Si^{4+} and Al^{3+} for the anisotropic zeolite ZSM-5 crystals

The coordination environments of Si and Al in the anisotropic zeolite ZSM-5 crystals were evaluated by ^{29}Si and ^{27}Al ssNMR, as shown in Figure S6 and Figure S7, respectively. The ^{29}Si magic angle spinning (MAS) ssNMR spectra of the zeolite samples and their deconvolution curves are shown in Figure S6.[6,25] Five peaks can be observed in both the *a*-oriented and *b*-oriented zeolite ZSM-5 crystals after deconvoluting with Gaussian curves. The peaks at ~ -116 and ~-112, ~ -108 and ~ -105, and ~ -102 ppm are assigned to Si(0Al), Si(1Al) and silanol groups, respectively. The deconvoluting results are listed in Table S3. It shows that the two zeolite crystals with different orientations have comparable Si/Al ratios with 32 and 34 for the *a*-oriented and *b*-oriented zeolite ZSM-5 crystals, respectively. Moreover, ^{27}Al ssNMR spectroscopy was used to elucidate the coordination environment of different Al species. As shown in Figure S7, two peaks are observed for both samples at ~ 56 and ~ 0 ppm, being assigned to the four-coordinated framework Al^{3+} and the six-coordinated extra-framework Al species, respectively. The intense peak at ~ 56 ppm and less intense peak at ~ 0 ppm (Figure S7) as well as similar peak ratios of four- to six-coordinated Al, show the predominance of framework Al^{3+} species and similar Al environment distributions in the two samples. Additionally, it has been demonstrated that the location of framework Al^{3+} is heavily determined by the locations of the structure directing agents (SDAs), and that the addition of inorganic mineralizers will modify the locations due to charge balance effect.[26,27] Considering the comparable structures of TPA^{+} and trimer-TPA^{3+} (Figure S1), we expect that the locations of framework Al^{3+} are also comparable in the anisotropic zeolite ZSM-5 crystals. Therefore, these results confirm that the effect of steric structure of zeolite channels on catalysis can be isolated by preparing samples with different morphologies.

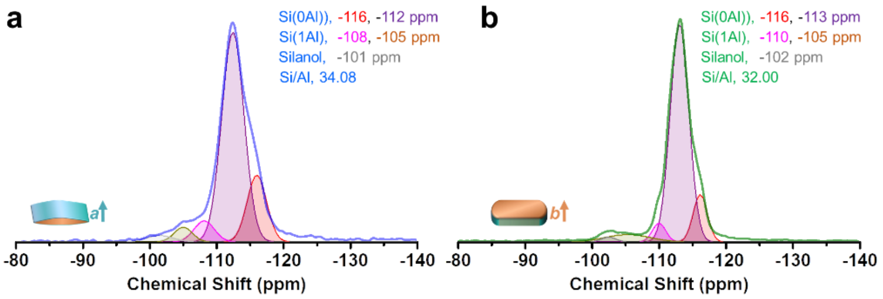

Figure S6. Direct-excitation ^{29}Si magic angle spinning (MAS) ssNMR spectra of the a) *a*-oriented and b) *b*-oriented zeolite ZSM-5 crystals. The spectra were deconvoluted using a Gaussian function. The Si-species are identified as Q4 (−112 and −116 ppm), Q3 (−106 ppm and -108 ppm) and Q2 (−102 ppm).

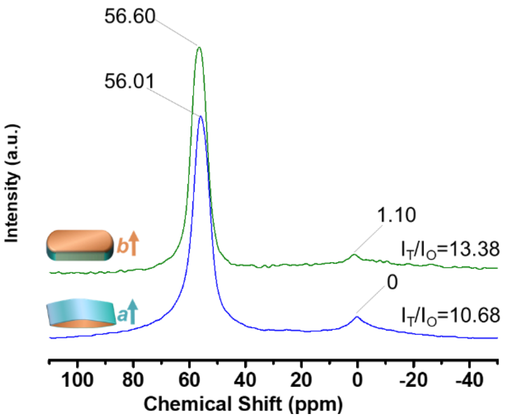

Figure S7. Normalized ^{27}Al direct excitation (DE) magic angle spinning (MAS) ssNMR spectra of the *a*-oriented (blue) and *b*-oriented (green) zeolite ZSM-5 crystals. The chemical shift values are reported for each peak.

Table S3. Parameters for the deconvolution of ^{29}Si direct excitation (DE) magic angle spinning (MAS) solid-state nuclear magnetic resonance (ssNMR) spectra of the *a*-oriented and *b*-oriented zeolite ZSM-5 crystals.

	***a*-ZSM-5**			***b*-ZSM-5**		
	Peak	Area	I (area %)	Peak	Area	I (area %)
Si(0Al)	-116	1.49E7	19.05	-116.15	1.29E7	12.50
Si(0Al)	-112.41	5.34E7	68.26	-113.05	7.76E7	75.19
Si(1Al)	-108.13	4.91E6	6.28	-109.99	4.82E6	4.67
Si(1Al)	-105	3.07E6	3.92	-105.27	6.49E6	6.29
Si-(OSi)3(OH)	-101.49	1.95E6	2.49	-102.46	1.40E6	1.36
Si/Al	38.24			36.01		

S5.4. Validating reaction locations within the anisotropic zeolite ZSM-5 crystals

Initially, molecular dynamics (MD) simulation was applied to qualitatively study the diffusion behaviors of molecules within zeolite channels with different dimensions. The diffusivity of the representative molecule, *i.e.,* methanol, within pure-Si zeolite ZSM-5 (MFI framework) was first calculated by fitting the mean squared displacement (MSD) curve (Figure S8).[28] The effect of morphology on the diffusion behaviors was further investigated using the obtained diffusion coefficient in a 2Dimensional (2D) model. The 2D model is constructed with the similar aspect ratio to the anisotropic zeolite ZSM-5 crystals. As discussed in the main text (Figure 1 and movie S1), the channels with short dimensions (SD) were preferentially filled with the molecules at the early stage of diffusion. Furthermore, no apparent diffusion of the molecules from the SD channels into the channels with long dimensions (LD) was observed, while diffusion within the SD channels was prompt at the same time. These results suggest that the molecules will enrich into the SD channels in the beginning of the reactions, although both channel dimensions are equivalently filled with the molecules eventually. Therefore, short time MTH reaction was performed over the as-synthesized anisotropic zeolite ZSM-5 crystals with different morphologies.

To prepare zeolite ZSM-5 crystals with HCP species primarily trapped into certain channel orientations, the MTH reaction was closely followed using *in situ* UV/Vis DRS.[4,29] We have demonstrated different reaction behaviors within the sinusoidal and straight zeolite ZSM-5 channels with the combination of *operando* UV/Vis DRS and uniformly oriented zeolite ZSM-5 thin-films.[4] Distinct UV/Vis DR spectra of HCP species were observed from the two different channel orientations. Specifically, HCP species with absorption band in the range of *ca.* 410-560 nm will primarily be formed in the straight channels during the MTH reactions.[4] Therefore, those species were used as a marker to locate the reactions within zeolite channels of the anisotropic zeolite ZSM-5 crystals. As shown in Figure 2, very low signal of UV/Vis absorbance in the range of *ca.* 410-560 nm was observed for the *a*-oriented zeolite ZSM-5 crystals during the first 2 min MTH reactions, suggesting that the reaction primarily occurs in the sinusoidal zeolite channels. Conversely, fast evolution and strong absorbance from HCP species were observed in the same range for the *b*-oriented analog during the first 2 min MTH reactions, showing that methanol molecules preferentially diffused into and were catalyzed primarily in the straight channels of the *b*-oriented zeolite ZSM-5 crystals.

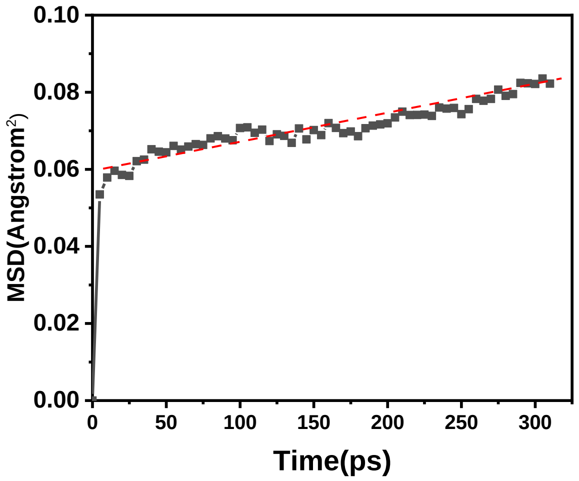

Figure S8. The mean squared displacement (MSD) profile of methanol in the pure-Si zeolite ZSM-5. The calculated diffusion coefficient D=1.11x10^{-13} m^2/s.

S5.5. 2D solid-state NMR spectra of the adsorbed methanol and dimethyl ether within the anisotropic zeolite ZSM-5 crystals

The binding structures of both the rigid and mobile methanol molecules (Figures S9-S10) were identified. In the experiments probing the rigid methanol, two different motifs, *i.e.,* side-on (η^2:η^2) and end-on (η^1:η^1) conformations, were found in both *a*-oriented and *b*-oriented zeolite crystals, indicating comparable adsorption environments in the two distinct crystals.[30] Moreover, the assignments are confirmed by examining the mobile molecules. As shown in Figures S9c-d, the intensity of the end-on conformation of methanol is enhanced in both zeolites. In contrast, the intensity of the side-on conformation is sharply decreased or absent, as in the *a*-oriented zeolite crystals. This demonstrates a lower mobility of the side-on conformers and a stronger H-bonding, suggesting a more rigid nature in the *a*-oriented zeolite crystals. Additionally, dimethyl ether (DME), a well-known intermediate of the MTH reaction, is observed in the ^{1}H-^{13}C correlation spectra for both anisotropic crystals (Figure S9).

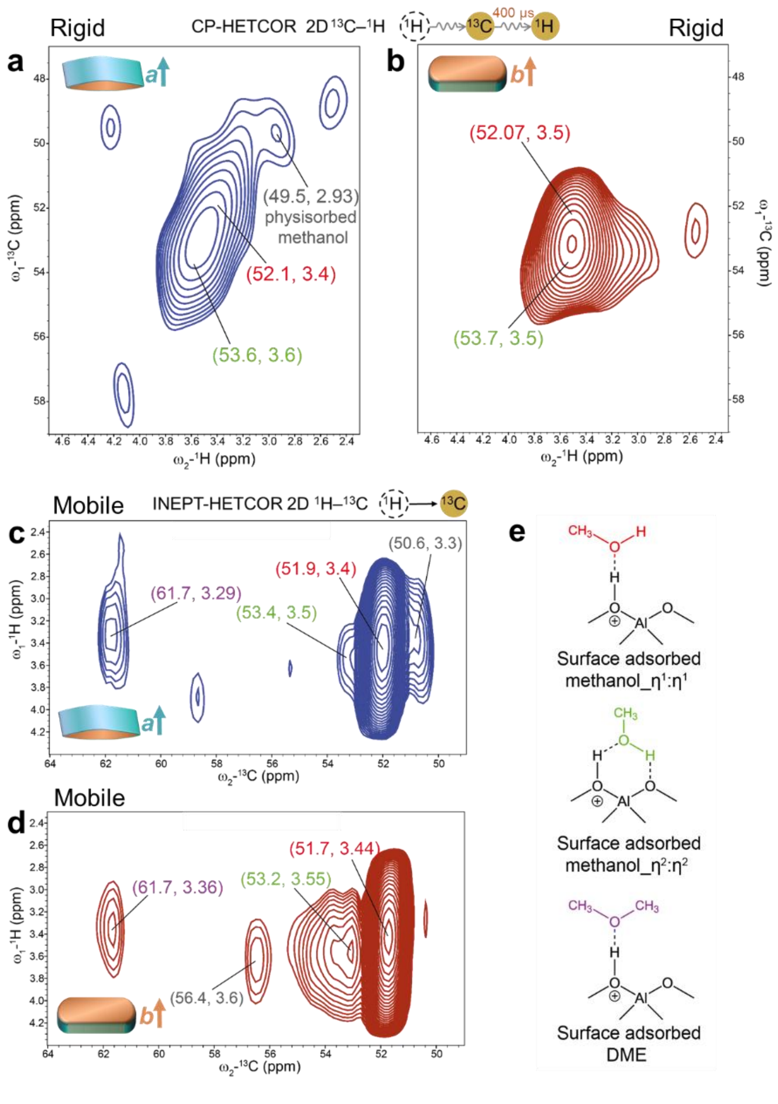

Figure S9. 2D magic angle spinning (MAS) ssNMR spectra of the surface adsorbed a,b) rigid and c,d) mobile methanol molecules in the anisotropic zeolite ZSM-5 crystals after 2 min of the methanol-to-hydrocarbons (MTH) at 623 K. a, c) and b,d) are the ssNMR spectra for the adsorbed molecules trapped in the *a*-oriented and *b*-oriented zeolite ZSM-5 zeolites, respectively. e) Overview of the surface adsorbed molecules. A contact time of 400 µs was used for the ^{13}C-^{1}H correlation experiment. All ssNMR spectra were recorded at 290 K, using 16 kHz MAS. Compared with the mobile reactant, the

chemical shifts of surface adsorbed methanol are all shifted downfield, indicating that the zeolite channels altered the electronic state of the immobilized methanol.[22]

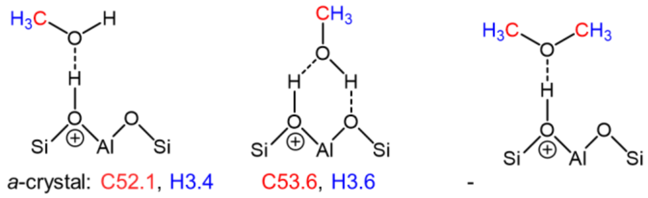

Rigid	*a*-crystal:	C52.1, H3.4	C53.6, H3.6	-
	b-crystal:	C52.07, H3.5	C53.6, H3.6	-
Mobile	*a*-crystal:	C51.9, H3.4	C53.4, H3.5	C61.7, H3.3
	b-crystal:	C51.7, H3.4	C53.2, H3.6	C61.7, H3.4

Figure S10. Overview of the adsorbed methanol and dimethyl ether detected from the ssNMR experiments along with their assigned chemical shifts.

S5.6. 2D ^{13}C-^{13}C spin-diffusion ssNMR spectra of the rigid molecules within the anisotropic zeolite ZSM-5 crystals using different mixing times

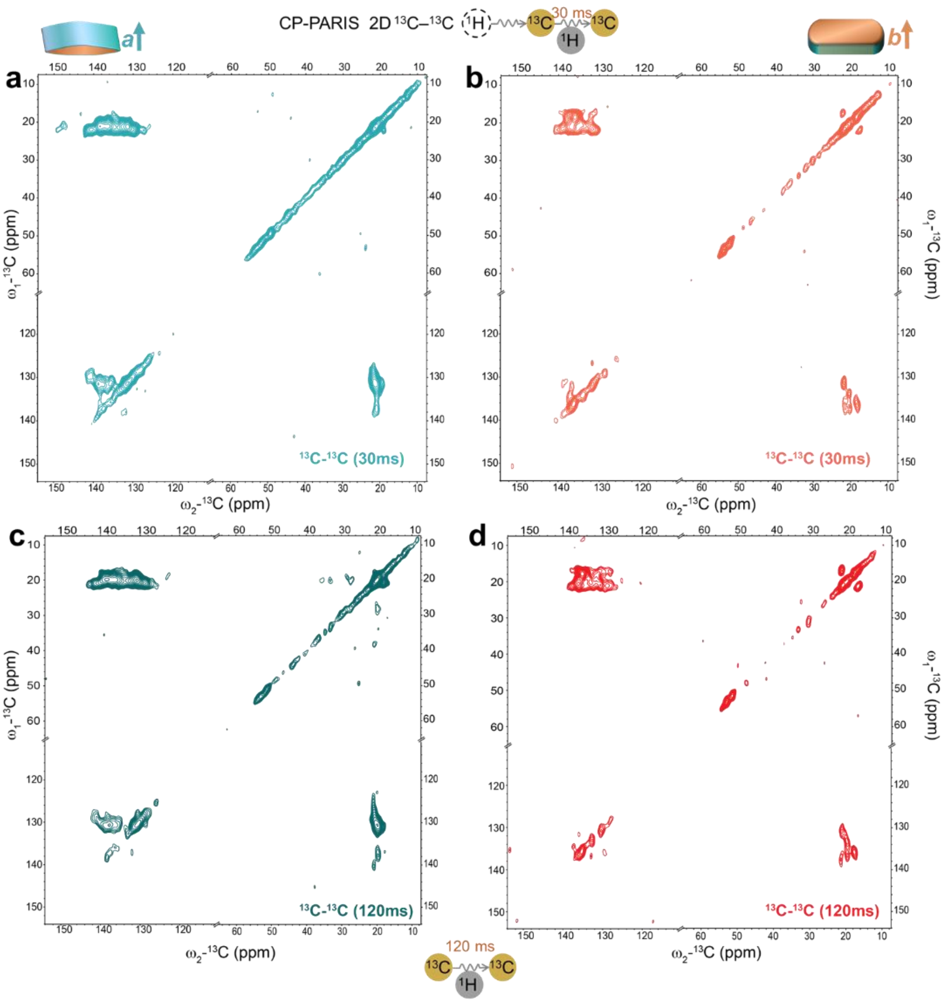

Figure S11. 2D ^{13}C-^{13}C magic angle spinning (MAS) ssNMR spectra of the rigid molecules in the a,c) *a*-oriented and b,d) *b*-oriented zeolite ZSM-5 crystals after 2 min of the methanol-to-hydrocarbons (MTH) at 623 K. The spectra were recorded at 290 K, using 16 kHz MAS. For the ^{13}C-^{13}C correlation experiment the polarization of ^{13}C atoms was achieved through cross-polarization (CP). A a,b) 30ms or c,d) 120ms phase-alternated recoupling irradiation scheme (PARIS) mixing period was used. Note that even with a longer mixing time (120ms) the cross-peaks between ~18 ppm and ~21 ppm resonances are absent in the *a*-oriented zeolite crystals. Instead, the cross-peaks become more prominent in the *b*-oriented zeolite crystals at a longer ^{13}C-^{13}C mixing, therefore confirming the presence of asymmetric methylated aromatics in the sample with a higher accessibility for the straight zeolite channels.

S5.7. Zoom-in 2D ^{13}C-^{13}C correlation ssNMR spectra of the rigid methylated aromatics molecules and overview of the reaction intermediates detected within the anisotropic zeolite ZSM-5 crystals

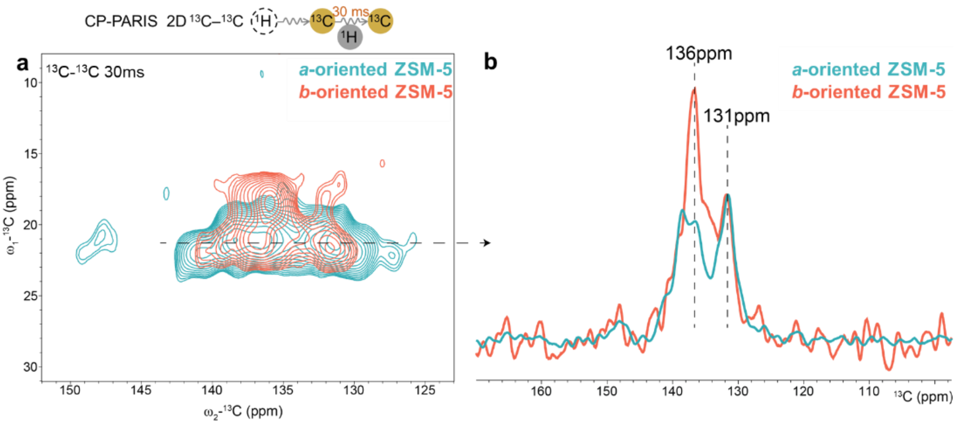

Figure S12. a) Zoom-in of the 2D ^{13}C-^{13}C magic angle spinning (MAS) ssNMR spectra of the rigid methylated aromatics molecules in the *a*-oriented and *b*-oriented zeolite ZSM-5 crystals after 2 min of the methanol-to-hydrocarbons (MTH) at 623 K. b) 1D projection (as defined in topspin) of the aromatic resonances in the direct dimension. The projections are normalized to the 131 ppm resonance. The spectra were recorded at 290 K, using 16 kHz MAS. The polarization of ^{13}C atoms was achieved through cross-polarization (CP), and a 30 ms phase-alternated recoupling irradiation scheme (PARIS) mixing period was used.

S5.8. 2D scalar-based ^{1}H-^{13}C and ^{13}C-^{13}C ssNMR spectra of mobile molecules within the anisotropic zeolite ZSM-5 crystals

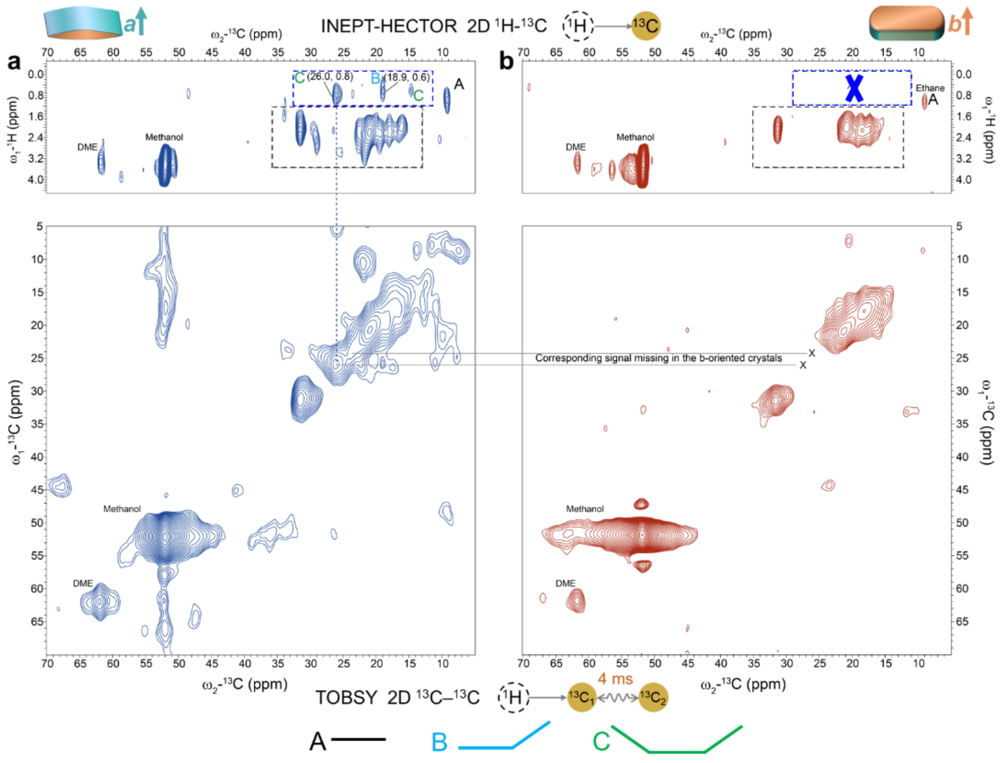

Figure S13. 2D scalar-based ^{1}H-^{13}C (top) and ^{13}C-^{13}C (bottom) magic angle spinning (MAS) ssNMR spectra of the mobile molecules trapped in the a) *a*-oriented and b) *b*-oriented zeolite ZSM-5 crystals**.** The black box indicates the mobile methyl resonances, which are equivalent to the immobile species shown in the right panels of Figure 4. The blue box highlights the hydrogen-transferred species only present in the *a*-oriented ZSM-5 crystals. For the ^{13}C-^{13}C correlation experiment a total through-bond correlation (TOBSY) mixing time of 4 ms was used.[16] The ^{1}H-^{13}C correlation spectra identified ethane at 9.15 ppm (^{13}C) and 0.98 ppm (^{1}H) as well as propane at 18.9 ppm (^{13}C) and 0.7 ppm (^{1}H) in the *a*-oriented zeolite ZSM-5 crystals, while only ethane (8.97(^{13}C) and 0.99 (^{1}H) ppm) was observed in the *b*-oriented analog.

S5.9. 2D scalar-based ^{13}C-^{1}H ssNMR spectra of the mobile molecules within the anisotropic zeolite ZSM-5 crystals

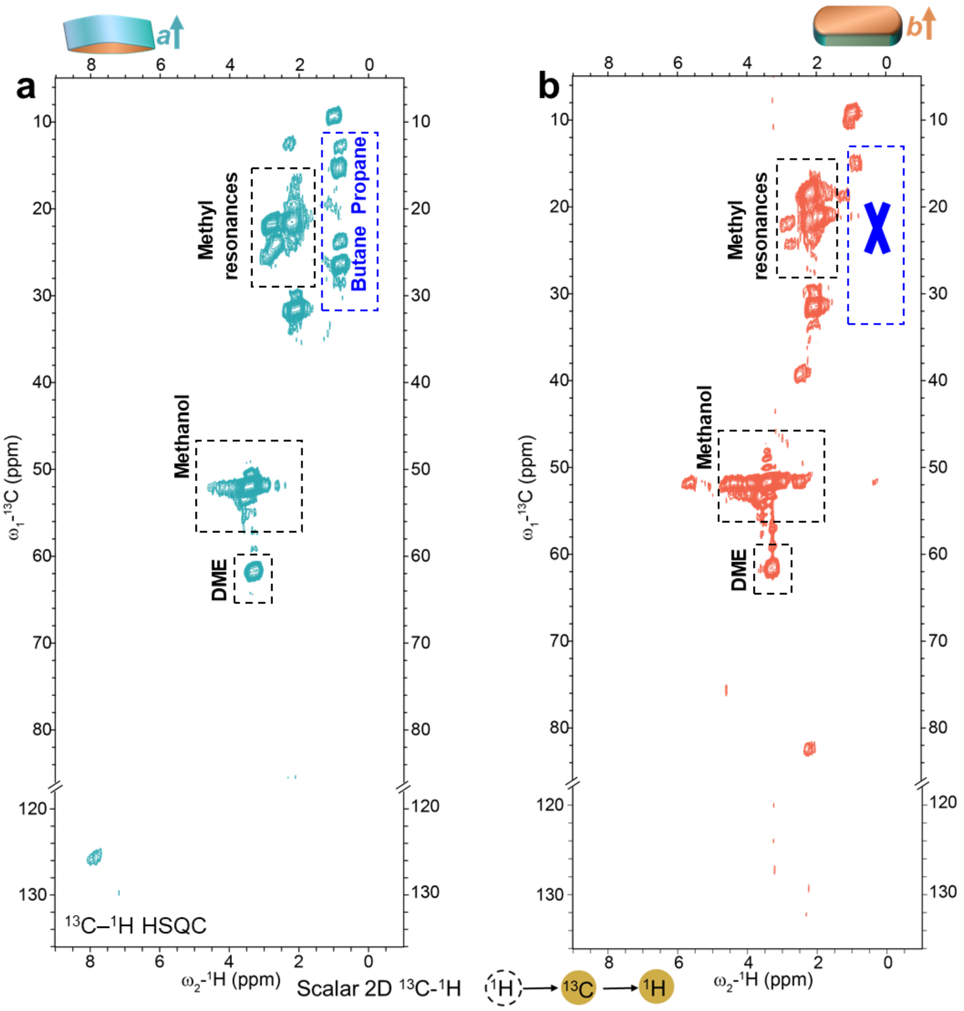

Figure S14. 2D scalar-based ^{13}C-^{1}H magic angle spinning (MAS) ssNMR spectra of the mobile molecules trapped in the a) *a*-oriented and b) *b*-oriented zeolite ZSM-5 crystals after 2 min of the methanol-to-hydrocarbons (MTH) at 623 K**.** The spectra were recorded at 290 K using 16 kHz MAS. The blue box highlights the hydrogen-transferred species only present in the *a*-oriented ZSM-5 crystals. The resonances at ~24 and 26 ppm (^{13}C) and 0.8 ppm (^{1}H) are only visible in the *a*-oriented crystals and are compatible with butane and/or tetramethylethane signal. Based on the literature and by combining the spectra of the scalar-based ^{13}C-^{1}H and ^{13}C-^{13}C correlations experiments (Figure S13), the signals were tentatively assigned to butane moieties.

S5.10. 2D dipolar-based ^{13}C-^{1}H correlation ssNMR spectra of the rigid molecules within the anisotropic zeolite ZSM-5 crystals

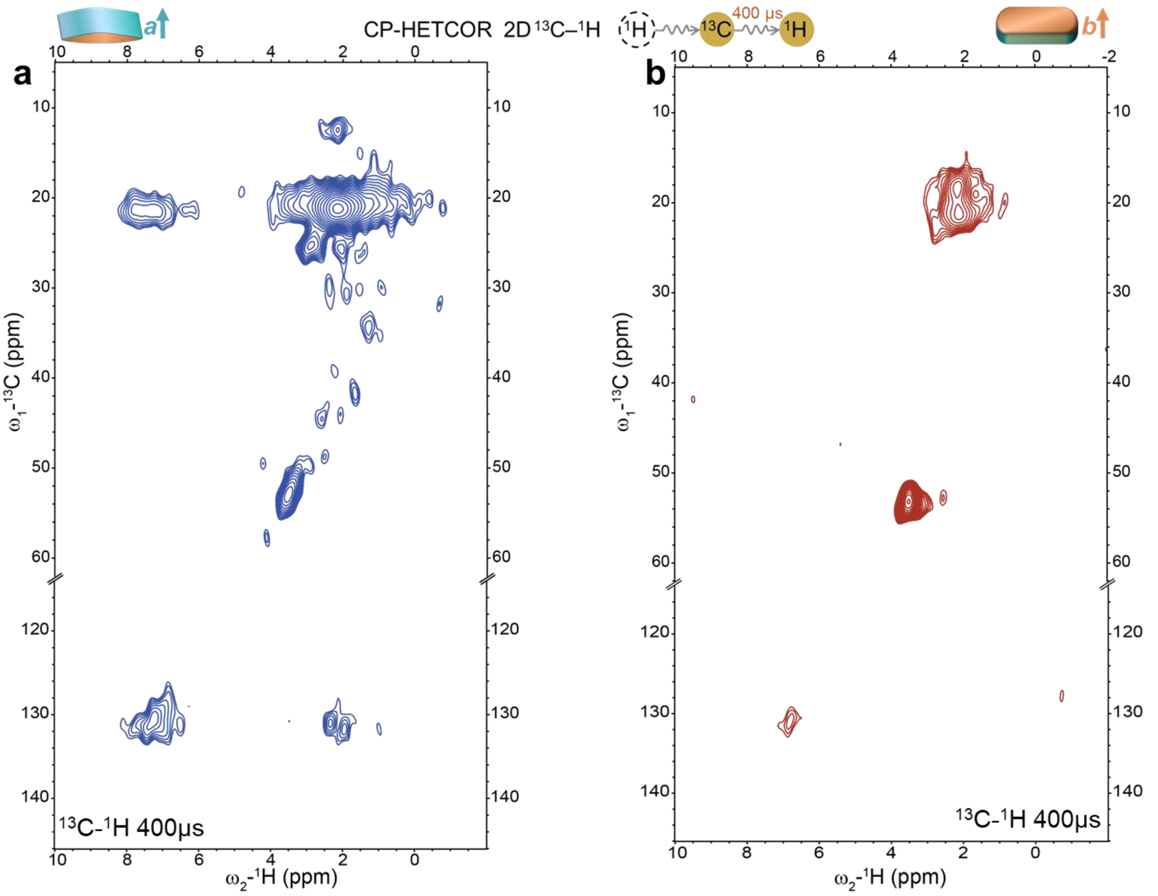

Figure S15. 2D dipolar (CP) based ^{13}C-^{1}H magic angle spinning (MAS) ssNMR spectra of the rigid molecules in the a) *a*-oriented and b) *b*-oriented zeolite ZSM-5 crystals after 2 min of the methanol-to-hydrocarbons (MTH) at 623 K. The spectra were recorded at 290 K, using 16 kHz MAS. A CP contact time of 400 μs was used for the ^{13}C-^{1}H transfer.

S5.11. Remaining unassigned ssNMR spin-systems for the anisotropic zeolite ZSM-5 crystals

Table S4. Remaining unassigned ssNMR spin-systems for the *a*-oriented zeolite ZSM-5 crystals. Overview of the unassigned ssNMR spin-systems from the reaction intermediates observed in this work. Data for purely mobile (denoted as M) and rigid (denoted as R) molecules/fragments are summarized. Species shown in the following spin-systems could not be assigned to a specific structure in a definitive manner, due to missing correlations and/or spectral crowding. The signals only visible in the *a*-oriented zeolite ZSM-5 crystals are indicated in blue.

Name	^{13}C1 [ppm]	^{1}H1 [ppm]	^{13}C2 [ppm]	^{1}H2 [ppm]	^{13}C3 [ppm]	^{1}H3 [ppm]
M11	125.6	7.87				
M1						
M2						
M3	21.4	2.23				
M4	19.4	2.23				
M5	17.8	2.12				
M6	16.1	1.99				
M8	21.7	2.88				
M9	24.05	2.68				
M10	23.8	0.79				
M12	12.85	0.80				
M13	15.2	0.74				
R1	21.9	1.95	140.6	7.84	129.6	
R2	21.17	2.32	138.5	7.29	131.2	
R3	16.7	2.13	135.7		131.3	
R4	23.2	2.91	131.5			
R5	34.3	1.30				
R6	41.9	1.66				

Table S5. Remaining unassigned ssNMR spin-systems for the *b*-oriented zeolite ZSM-5 crystals. Overview of the unassigned ssNMR spin-systems from the reaction intermediates observed in this work. Data for purely mobile (denoted as M) and rigid (denoted as R) molecules/fragments are summarized. Species shown in the following spin-systems could not be assigned to a specific structure in a definitive manner, due to missing correlations and/or spectral crowding. The signals only visible in the *b*-oriented zeolite are indicated in orange.

Name	^{13}C1 [ppm]	^{1}H1 [ppm]	^{13}C2 [ppm]	^{1}H2 [ppm]	^{13}C3 [ppm]	^{1}H3 [ppm]	^{13}C4 [ppm]	^{1}H4 [ppm]
M12	82.4	2.18						
M1	31.4	2.09						
M2	21.0	2.01						
M3	18.4	2.16						
M4	17.2	2.00						
M6	14.9	0.85						
M7	23.8	2.05		2.79				
M8	22.0	2.04		2.77				
M9	29.9	2.15						
M10	39.2	2.49						
R1	18.4	2.13	22.5		136.1			
R2	21.7	2.15	133.8	2.10	135.9		139.1	
R3	20.3	2.11	136.6		133.5			

S6. References

[1] J. Choi, S. Ghosh, Z. Lai, M. Tsapatsis, *Angew. Chem. Int. Ed.* **2006**, *45*, 1154–1158.
[2] Y. Shen, T. T. Le, D. Fu, J. E. Schmidt, M. Filez, B. M. Weckhuysen, J. D. Rimer, *ACS Catal.* **2018**, *8*, 11042–11053.
[3] N. Wang, W. Sun, Y. Hou, B. Ge, L. Hu, J. Nie, W. Qian, F. Wei, *J. Catal.* **2018**, *360*, 89–96.
[4] D. Fu, O. van der Heijden, K. Stanciakova, J. E. Schmidt, B. M. Weckhuysen, *Angew. Chem. Int. Ed.* **2020**, *10.1002/anie.201916596.*
[5] I. Yarulina, K. D. Wispelaere, S. Bailleul, J. Goetze, M. Radersma, E. Abou-Hamad, I. Vollmer, M. Goesten, B. Mezari, E. J. M. Hensen, J. S. Martínez-Espín, M. Morten, S. Mitchell, J. Perez-Ramirez, U. Olsbye, B. M. Weckhuysen, V. van Speybroeck, F. Kapteijn, J. Gascon, *Nat. Chem.* **2018**, *10*, 804–812.
[6] C. A. Fyfe, G. C. Gobbi, G. J. Kennedy, J. D. Graham, R. S. Ozubko, W. J. Murphy, A. Bothner-By, J. Dadok, A. S. Chesnick, *Zeolites* **1985**, *5*, 179–183.
[7] B. M. Fung, A. K. Khitrin, K. Ermolaev, *J. Magn. Reson.* **2000**, *142*, 97–101.
[8] A. Pines, M. G. Gibby, J. S. Waugh, *J. Chem. Phys.* **1973**, *59*, 569–590.
[9] G. A. Morris, R. Freeman, *J. Am. Chem. Soc.* **1979**, *101*, 760–762.
[10] D. H. Zhou, C. M. Rienstra, *J Magn Reson* **2008**, *192*, 167–172.
[11] M. Weingarth, P. Tekely, G. Bodenhausen, *Chem. Phys. Lett.* **2008**, *466*, 247–251.
[12] M. Weingarth, D. E. Demco, G. Bodenhausen, P. Tekely, *Chem. Phys. Lett.* **2009**, *469*, 342–348.
[13] G. Bodenhausen, D. J. Ruben, *Chem. Phys. Lett.* **1980**, *69*, 185–189.
[14] A. J. Shaka, P. B. Barker, R. Freeman, *J. Magn. Reson.* **1985**, *64*, 547–552.
[15] A. J. Shaka, J. Keeler, T. Frenkiel, R. Freeman, *J. Magn. Reson.* **1983**, *52*, 335–338.
[16] M. Baldus, B. H. Meier, *J. Magn. Reson.* **1996**, *121*, 65–69.
[17] J. Haber, J. H. Block, B. Delmon, *Pure Appl. Chem.* **1995**, *67*, 1257–1306.
[18] A. Lesage, M. Lelli, D. Gajan, M. A. Caporini, V. Vitzthum, P. Miéville, J. Alauzun, A. Roussey, C. Thieuleux, A. Mehdi, G. Bodenhausen, C. Copéret, L. Emsley, *J. Am. Chem. Soc.* **2010**, *132*, 15459–15461.
[19] Y. Matsuki, T. Maly, O. Ouari, H. Karoui, F. Le Moigne, E. Rizzato, S. Lyubenova, J. Herzfeld, T. Prisner, P. Tordo, R. G. Griffin, *Angew. Chem. Int. Ed.* **2009**, *48*, 4996–5000.
[20] D. Mance, J. van der Zwan, M. E. Z. Velthoen, F. Meirer, B. M. Weckhuysen, M. Baldus, E. T. C. Vogt, *Chem. Commun.* **2017**, *53*, 3933–3936.
[21] W. Lee, M. Tonelli, J. L. Markley, *Bioinformatics* **2015**, *31*, 1325–1327.
[22] A. D. Chowdhury, K. Houben, G. T. Whiting, S.-H. Chung, M. Baldus, B. M. Weckhuysen, *Nat. Catal.* **2018**, *1*, 23–31.
[23] A. D. Chowdhury, A. Lucini Paioni, K. Houben, G. T. Whiting, M. Baldus, B. M. Weckhuysen, *Angew. Chem. Int. Ed.* **2018**, *57*, 8095–8099.
[24] A. D. Chowdhury, I. Yarulina, E. Abou-Hamad, A. Gurinov, J. Gascon, *Chem. Sci.* **2019**, *10*, 8946–8954.
[25] H. Wang, L. Su, J. Zhuang, D. Tan, Y. Xu, X. Bao, *J. Phys. Chem. B* **2003**, *107*, 12964–12972.
[26] T. Yokoi, H. Mochizuki, S. Namba, J. N. Kondo, T. Tatsumi, *J. Phys. Chem. C* **2015**, *119*, 15303–15315.
[27] G. Ricchiardi, J. M. Newsam, *J. Phys. Chem. B* **1997**, *101*, 9943–9950.
[28] D. Frenkel, B. Smit, *Understanding Molecular Simulation: From Algorithms to Applications*, Elsevier, **2001**.
[29] D. Mores, J. Kornatowski, U. Olsbye, B. M. Weckhuysen, *Chem. Eur. J.* **2011**, *17*, 2874–2884.
[30] A. D. Chowdhury, A. Lucini Paioni, G. T. Whiting, D. Fu, M. Baldus, B. M. Weckhuysen, *Angew. Chem. Int. Ed.* **2019**, *58*, 3908–3912.
